# Disease in the Society: Infectious Cadavers Result in Collapse of Ant Sub-Colonies

**DOI:** 10.1371/journal.pone.0160820

**Published:** 2016-08-16

**Authors:** Raquel G. Loreto, David P. Hughes

**Affiliations:** 1 Department of Entomology and Center for Infectious Disease Dynamics, Pennsylvania State University, University Park, 16802 Pennsylvania, United States of America; 2 CAPES Foundation, Ministry of Education of Brazil, Brasília 70040–020 DF, Brazil; 3 Department of Biology, Pennsylvania State University, University Park, 16802 Pennsylvania, United States of America; Universidade de Sao Paulo Faculdade de Filosofia Ciencias e Letras de Ribeirao Preto, BRAZIL

## Abstract

Despite the growing number of experimental studies on mechanisms of social immunity in ant societies, little is known about how social behavior relates to disease progression within the nests of ants. In fact, when empirically studying disease in ant societies, it is common to remove dead ants from experiments to confirm infection by the studied parasite. This unfortunately does not allow disease to progress within the nest as it may be assumed would happen under natural conditions. Therefore, the approach taken so far has resulted in a limited knowledge of diseases dynamics within the nest environment. Here we introduced a single infectious cadaver killed by the fungus *Beauveria bassiana* into small nests of the ant *Camponotus castaneus*. We then observed the natural progression of the disease by not removing the corpses of the ants that died following the first entry of the disease. Because some behaviors such as social isolation of sick individuals or the removal of cadavers by nestmates are considered social immune functions and thus adaptations at the colony level that reduce disease spread, we also experimentally confined some sub-colonies to one or two chamber nests to prevent the expression of such behaviors. Based on 51 small nests and survival studies in 1,003 ants we found that a single introduced infectious cadaver was able to transmit within the nest, and social immunity did not prevent the collapse of the small sub-colonies here tested. This was true whether ants did or did not have the option to remove the infectious cadaver. Therefore, we found no evidence that the typically studied social immunity behaviors can reduce disease spread in the conditions here tested.

## Introduction

The social insects (wasps, bees, ants and termites) are the dominant fauna in most terrestrial habitats [1–3] and, as such, are hosts to many parasitic life forms [4]. The high density of individuals and the close confines of typical nests, together with the elevated degree of relatedness within full and half-sibling groups, raises the possibility that such societies are ideal for the rapid spread of infectious agents. The recent increase in colony collapse disorder in honeybees (*Apis mellifera*) shows that such societies can collapse and this can occur across multiple years [5, 6]. Although the presence of parasites is seen as an important factor involved in colony collapse disorder in the honeybee [7–9], other factors such as pesticides and other human mediated stress (e.g. habitat loss) are suspected to also play a significant role [10, 11]. Thus, while honeybees show that insect societies can collapse, it is generally assumed that human practices are strongly influencing the colony collapse in bees [10, 12].

In naturally occurring insect societies—distinct from the managed honeybee—the absence of outbreaks, despite the presence of parasites, is somewhat of a paradox. The apparent rarity of collapse due to diseases in natural societies is often explained by the social immunity hypothesis [13]. It predicts that, in addition to individual immunity, the social insects can collectively behave, resulting in prevention and control of parasite transmission within the colony. Support for this hypothesis comes from extensive laboratory studies which have demonstrated that workers behave differently in the presence of a potential source of infections [4, 14–18]. These altered behaviors are then considered adaptive and are suggested to prevent the spread of the infectious agent and the ultimate collapse of the colony [13].

Social immunity in ants is well studied [19]. This reflects the importance of ants which make up over 50% of the biomass in many habitats such as tropical forests [20], and play a crucial role in these ecosystems [21]. Unlike wasps and bees, worker ants cannot fly and must walk over a variety of substrates to forage for food. This likely exposes them to microbial parasites commonly found in the soil, such as entomopathogenic fungi [22–24]. The likely elevated encounter rate with parasites makes the ants good models to explore social immunity.

The literature strongly supports the contention that ant social behavior is adaptive and reduces the cost of parasites. It describes how the use of venom [25], the collection of resin from plants [26, 27], collective grooming [28–30] and ‘vaccination’ behavior [31] are employed to diminish the threat of infectious diseases. However, despite many studies of ant behavior in disease contexts, there is no causal evidence that the expression of social immunity results in increased survival at colony level. For example, Jaccoud et al. [32] observed the epizootic of a parasitic fungus experimentally applied in the foraging arena of the leaf-cutting ant *Atta sexdens rubropilosa*. Although they reported collective behavior, such as biohazard isolation and elevated grooming rates, it did not result in the survival of the colony. Therefore, it remains unclear if social immunity is, in fact, effective at that colony level and actually results in the prevention of colony collapse.

One difficulty in establishing this link is the methodology commonly employed. Studies on social immunity often follow standard experimentation protocols and, although these are important to answer certain questions, they do not account for other relevant aspects. For example, studies often use orders of magnitude more spores than might be found in the soil and apply that as a topical, liquid solution in highly reduced settings such as plastic cages [19]. Therefore, the approach taken so far may in fact limit our understanding of the relationship between social behavior and disease progression. Most of published studies on ant social immunity employed two common parasites which are the generalist entomopathogenic fungi *Beauveria bassiana* and *Metarhizium anisopliae* [19]. In natural conditions, ants may come across scattered conidia (propagules of the fungus) in the soil at low loads [22, 23]. In experimental studies, the most common exposure route is the topical application of highly concentrated fungal conidia solution direct onto the ant body [19]. Some exceptions are when the solution of conidia is applied in the food [18] or filter paper, on which ants are placed to walk over [27, 33, 34]. It is possible that, in nature, the most intense exposure to generalist fungal parasites is when the ants encounter infectious cadavers. These cadavers produce a high amount of conidia [35, 36] and we hypothesized that the cost-benefit relationship of the collective behavior in this situation may be different from the conditions that are typically established for experimental assays. Only two studies have used infectious cadavers as a route of exposure [35, 37]. As such, we lack an understanding of how infectious cadavers could impact the colony and its behavior.

Another standard practice of these studies is to immediately remove the dead ants from the nest and incubate the cadaver to allow the fungus to grow out from the body [32, 33, 35, 38, 39]. Cadaver removal is considered an important practice to confirm that the death is in fact due to the fungal infection and not some other cause. However, the removal of cadavers interrupts the infection chain, thereby preventing the disease from progressing within the nest as may happen under natural conditions. An exception was an assay performed by Stimac and Pereira [37], which aimed to study the transmission of *B*. *bassiana* in real size colonies, by mixing live infected ants or infectious cadavers with healthy live ants. Although they observed up to 98% mortality (depending on the experiment), they merely assumed the mortality was due to transmission and did not confirm the infection of the individual cadavers. It is not an overstatement to then say that, despite extensive studies on social immunity, we do not know how infectious diseases progress within the nest, after an initial exposure.

Here we study the progression of a generalist fungal parasite following the introduction of infectious cadavers into sub-colonies of ants. We designed plaster nests to mirror natural conditions and allow the generalist enthomopathogenic fungus *Beauveria bassiana* to grow and produce conidia from cadavers of *Camponotus castaneus* within the nest. These cadavers were not removed during the experiment (28 days). Because nest architecture shapes collective behavior [40] and space segregation is presumably important for avoiding infections [13, 41, 42], we tested three different nest architectures. We focused on small sub-colonies of ants (20 workers) as this reflects the condition inside the nest chamber of a large colony, as well as the initial stage of colony life, which is considered susceptible to perturbation. Our work identifies important lacunae in our understanding of disease in ant societies by describing the infection chain within a small nest. In our opinion, this is the first step to a more complete understanding of the link between collective immune behavior and the eventual success or failure of disease progression within ant nests.

## Material and Methods

Colonies of the carpenter ant *Camponotus castaneus* were collected in Donald County, South Carolina (34.332110, -82.387131), during the summer of 2014. This a private patch of temperate deciduous forest and we received permission from the owner to collect ants in this area. They were kept in the laboratory on a 12:12h light cycle, 25°C and were fed *ad libitum* on 20% sugar-water, water and crickets. As a source of infection, we used the generalist entomopathogenic fungus *Beauveria bassiana* that effectively infects and kills ants in laboratory setting. Although it is likely not important for natural populations of ants, we chose this pathogen because it is frequently used for studies in ants [19]. We used the strain I93-825, originally isolated from a coleopteran specimen, provided by Dr. Nina Jenkins, Department of Entomology, The Pennsylvania State University.

In order to infect new hosts, the fungus first needs to grow from the cadaver of its current host and produce conidia. For this reason, we opted for plaster nests, which allows the fungus to develop after the ant dies and produce the infective propagules (S1 Fig). To study the effect of nest architecture on the progression of the parasite and the survival of ants, we created three nest architectures: (a) one closed chamber (OCC), where 20 ants could not leave the chamber (S2A Fig); (b) two connected closed chambers (TCC), where 20 ants could freely move between the two chambers, but could not leave the nest (S2B Fig); (c) two connected open chambers (TOC), where 20 ants could freely move between the chamber, as well as leave the nest (S2C Fig) to an empty arena. All the nests were supplied with *ad libitum* sugar-water and water, which were provided by inserting tubes in the side of the chamber (S2 Fig). In the case of two chambers (TCC and TOC), the chamber with the feeding tubes is referred to as the ‘food chamber’. The TOC nests had the entrance on the opposite side of the food chamber (S2 Fig). The dimension of the chambers and the connection between them (when present) were 2.5W x 4.5L x 2.5H cm and 1W x 2L 2.5H cm, respectively. Note that the density of ants in OCC nests was twice the density of ants in TCC and TOC nests. The chambers were covered with plastic sheet punctured with pins (size N°5).

To obtain the infectious cadavers that were introduced into the nests, we treated filter paper with 1 ml of a conidia suspension at the concentration of 5 x 10^9^ per ml (0.05% Tween 80). We intentionally used a large amount of conidia to ensure the infection and death of the ants. Groups of 15 ants were kept in Petri dishes containing the treated filter paper for 48 hours. The ants were then placed in a clean cage and monitored daily. The dead ants were individually incubated (28°C) for four days to allow the fungus to grow and produce conidia, resulting in infectious cadavers. All the infectious cadavers originated from one colony, which was not used in the experiment. *Beauveria bassiana* infects more than 200 species of insects [43] and, under natural conditions, ants likely come across infectious cadavers of other insects. Thus, the colony of origin of the infectious cadaver is not relevant to this experiment. All the infectious cadavers were photographed before being introduced to the nest. For the controls, we opted for dead nestmates because ants are more like to encounter non-infectious dead nestmates more often than they encounter cadavers of other insects. Thus, we froze healthy nestmates, from their respective colonies, by placing them in the -80°C freezer for 5 minutes. These are called ‘control cadavers’ throughout. The control cadavers were allowed to air dry for 10 minutes before being used.

The live ants were housed in the plaster nests and allowed to acclimatize for 48 hours. During the acclimation period, the ants in the TCC and TOC nests were restricted to the food chamber, not having access to the second chamber (S2B and S2C Fig). Consequently, ants in the TOC nests were not able to leave the nest during the acclimation period, since the nest entrance was opposite to the food chamber (S2C Fig). After 48 hours of acclimation in the plaster nests, the ants were anesthetized by placing the entire nest in the refrigerator (9°C) for 10 minutes. For OCC nests, the infectious cadaver was introduced in the center of the chamber (S2A Fig). For TCC and TOC nests, we first opened the access to the second chamber before introducing the cadaver in the center of the food chamber (S2B and S2C Fig). In each case, the cadavers were carefully placed in the chamber by the use of Featherweight forceps (Bioquip). The introduced cadavers were removed after 24 hours, using the same anesthetizing procedure. Following the removal, the all the introduced cadavers were photographed.

The survival of the ants was monitored daily for the 28 days following cadaver removal. Because our goal was to study the disease dynamics inside the nest, none of the ants that died after the exposure to the introduced cadavers were removed from the nests. Ants that died after the initial exposure are termed ‘secondary cadavers’. During the 28 days of the experiment, we carefully checked for fungal growth on secondary cadavers without disturbing the nests. For the treatments with two chambers, we also recorded the location of live and dead ants (i.e. in the food chamber, the second chamber or outside the nest in the case of the open nests). At the end of the experiment (day 28), any secondary cadavers that did not display fungal growth were incubated for confirmation of the infection. Ants that were still alive were monitored and incubated postmortem.

A total of 16 different colonies were exposed to cadavers in this experiment. Due the variable number of ants in the source colonies, not all colonies were exposed to both infectious and control cadavers using all types of nest architectures. Thus, for infectious cadavers, OCC, TCC and TOC were repeated with 16, 13 and 6 colonies, respectively. For controls, OCC, TCC and TOC were repeated with 8, 4 and 4 colonies, respectively (S1 Table). In total, we had 51 sub-colonies, consisting of 35 for infectious cadavers and 16 for control cadavers. Additionally, some nests had fewer or more than 20 ants (range 16–23; S1 Table). We performed a Cox proportional-hazard model to examine the effects of cadaver type and nest architecture on the survival of the ants. The model was built using cadaver type (levels: infectious or control) and nest architecture (levels: one closed, two closed or two open chambers) as explanatory co-variables for survival. The colony of origin was entered as a stratum, verifying the effect of the co-variables within each nest. We used Log-rank to determine difference among levels. In the TCC nest, to test if the ants and cadavers were randomly distributed among the food and second chambers, we use a general linear model with binomial distribution. All the statistical analyses were performed using R.

## Results

The fungal phenotype changed following the introduction of the infectious ant cadaver into the nest. Initially the cadaver of the ant had a profuse, fuzzy covering of fungal growth, which is characteristic of high conidial numbers (S3 Fig). Photographs of the introduced cadavers 24h after they were placed inside the nest revealed the disappearance of this characteristic phenotype of *B*. *bassiana* (S3 Fig). Out of 35 infectious cadavers introduced in the nests, 14 (40%) were broken up into pieces in less the 24 hours. In contrast, the majority of the control cadavers were left intact by the ants (14 out of 16 (87.5%)). In the two connected open chambers (TOC) nests, all (n = 6) the infectious cadavers were removed from the nest in less than two hours. In this case, only one (out of six) was broken up in pieces. Most of the control cadavers (five out of six) were found outside the nest after 24 hours, but the removal did not happen within the first 2 hours (the time period that we observed for).

In the one closed chamber (OCC) nests exposed to the fungus, where the removal of the secondary cadavers (i.e. dead after expose to introduced cadavers) was not an option, the cadavers were piled up in a corner of the chamber, usually in opposite side to where the live ants were (S4A Fig). In the two connected closed chambers (TCC) nests, we made daily observations on the location of live ants and the secondary cadavers. The live ants and secondary cadavers were not randomly distributed between the two chambers (z = 2.218, p = 0.02). On the day when 50% of the ants in the sub-colonies were dead, only 1 out of the 13 nests had live ants and cadavers mixed in both chambers (Flem11_TCC). The remaining 12 TCC nests had live ants and secondary cadavers occupying separate chambers (S4B Fig). Although we did not take specific measurements, spatial segregation between live and dead ants was also observed in the TOC nests, with all the secondary cadavers being removed from the nest and left outside, in the plastic cage.

**Fig 1:**
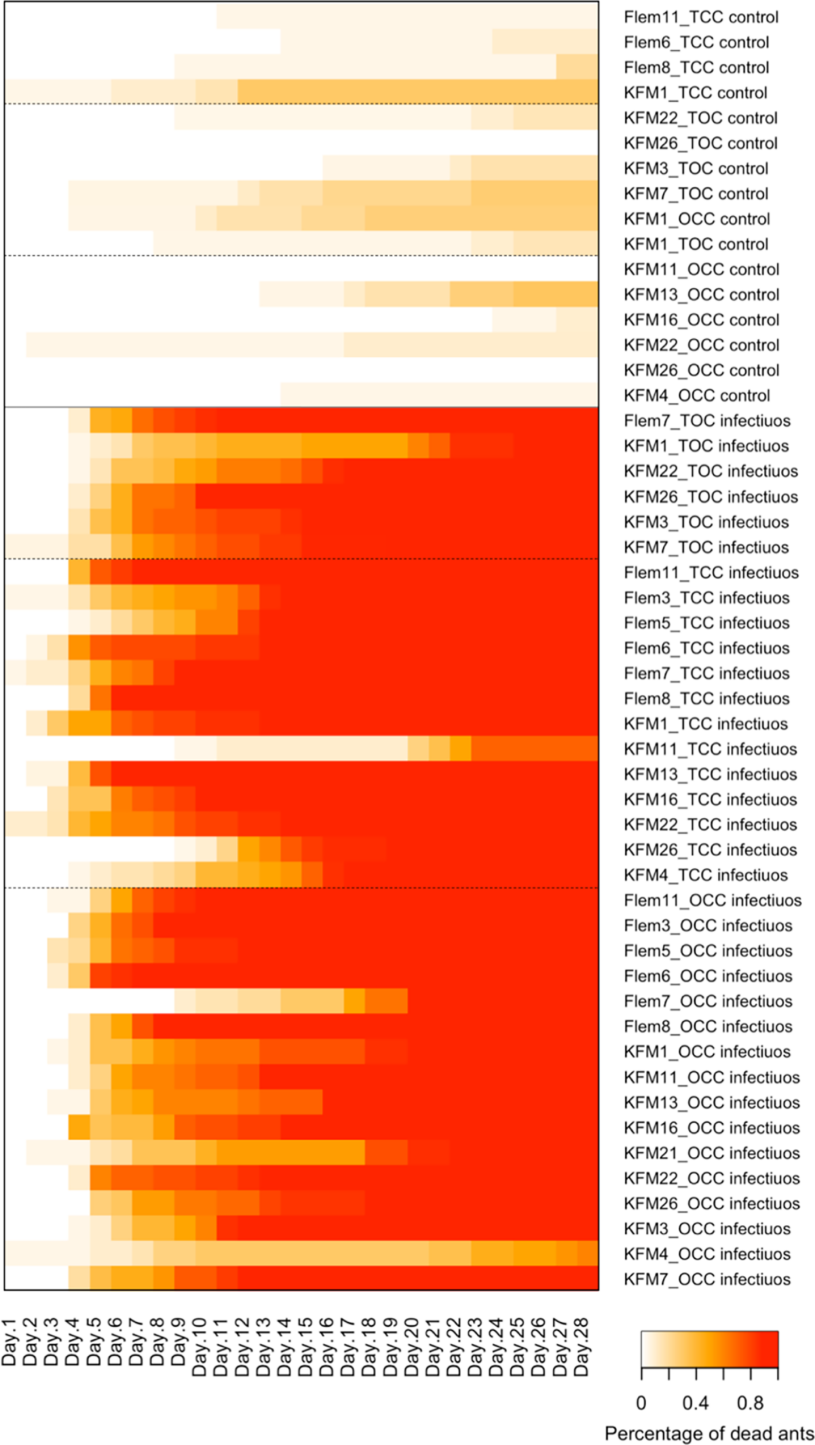
Fungal disease progression within small sub-colonies of ants. The percentage of dead ants across 28 days of experiment. White means all ants are alive and red means all ants have died.

The survival of 1,003 ants was analyzed in this study, of which 681 were exposed to infectious and 321 to control cadavers (S2 Table). The exposure to the pathogenic fungus *B*. *bassiana* via infectious cadavers decreased the survival of the ants when compared to exposure to control cadavers (Wald = 290.90, df = 5, p<0.0001) (Fig 1 and S5 Fig). The exposure to infectious cadavers increases the daily hazard of death to 97.3% when compared to controls. From the 692 ants exposed to the infectious cadaver, 681 (98%) died within the 28 days the experiment was run for. The 11 ants that did not die within 28 days were from 2 nests (KFM4 OCC (8) and Flem11 TCC (3)), and *B*. *bassiana* growth was observed post-incubation for all them (i.e. after they died). From the 311 ants exposed to control cadavers, only 45 (14%) of ants died and none of them had *B*. *bassiana* growth. The architecture of the nest had no effect on the survival of the ants (Wald = 290.90, df = 5, p = 0.5000) (Fig 1 and S5 Fig). No interaction was observed between treatment and nest architecture (*χ*^2^ = 1.639, df = 4, p = 0.8020).

**Fig 2:**
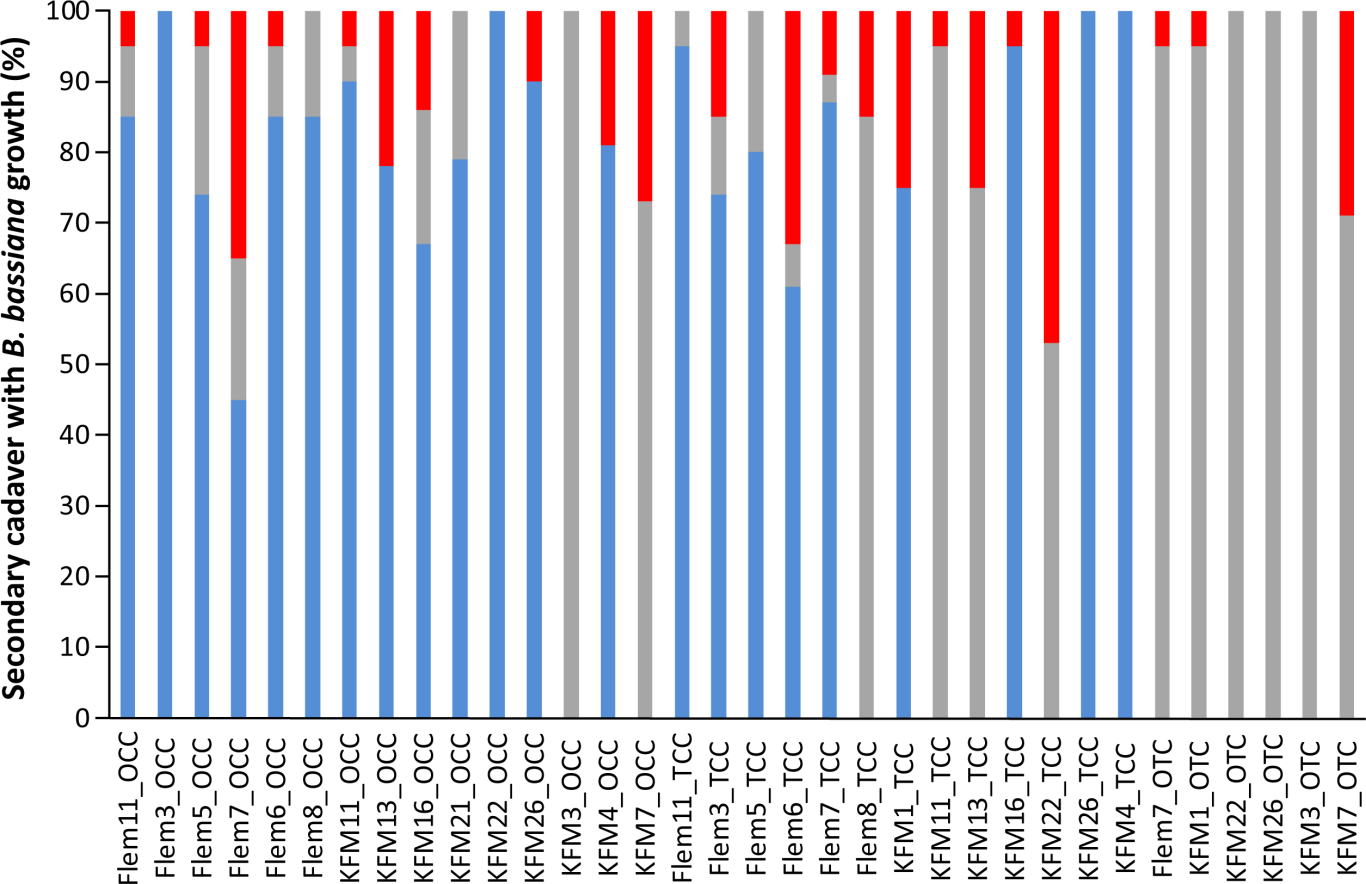
Percentage of secondary cadavers with *B*. *bassiana* growth. The blue bars correspond to secondary cadavers that displayed growth prior the end of the experiment (28 days). The grey bars represent the secondary cadavers that displayed growth post-incubation (after the end of the experiment >28 days). The red bars represent the secondary cadavers or live ants, from which the fungus was not recovered. The majority (86%) of ants were infected by *B*. *bassiana*. Note that the nest KFM1_OCC is not included in this figure due the bacterial growth over the cadavers.

It is important to establish if the ants that died during the experiment were actually infected by fungi, thus supporting the conclusion that the reason for their death was parasitism. For one colony (KFM1_OCC) we could not confirm infection due to the presence of a bacterial growth on the ants following their death. For remaining 34 nests exposed to infectious cadavers, 584 (86%) out of 672 secondary cadavers were confirmed for infection (Fig 2). The percent of infected ants/sub-colony was variable, with the lowest percent being 50 infected (1 nest had 50% infection) and highest percent being 100 (8 nests had 100% infection) (Fig 2). From the 584 infected ants we found 440 (64%) displayed fungal growth during the 28 days of experiment, while they were still inside the nest. A total of 232 ants were incubated post experiment, and 144 showed *B*. *bassiana* growth (Fig 2).

**Fig 3:**
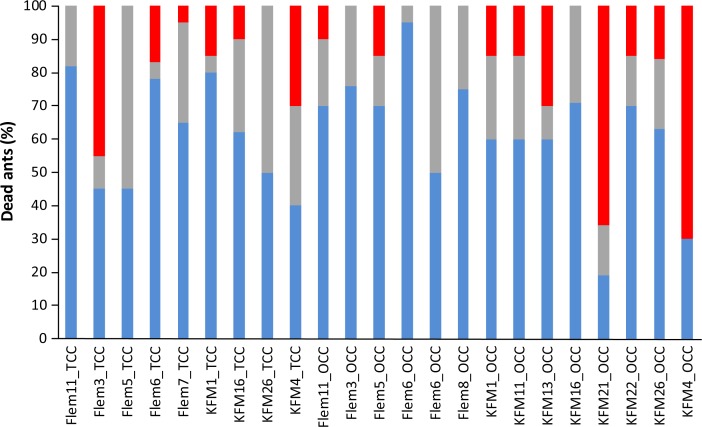
Estimation of percentage of ants infected by the introduced infectious cadavers. Blue bar corresponds to the percentage of ants already dead when we observed *B*. *bassiana* growth from secondary cadavers. These must have been infected by the introduced infectious cadavers. Gray bar corresponds to the percentage of ants that died after *B*. *bassiana* growth on the secondary cadavers, but before the minimal time required for the fungus to kill (time that the first ant died after exposure to the introduced cadaver). Red bar corresponds to the percentage of ants that could have been infected by either the introduced cadaver or, potentially, secondary cadavers. Note that, in this graphic, we only show the nests where fungus grew from secondary cadavers before 100% of the ants died.

In our experiment we initiated the infection with the introduced cadavers, but it is also possible some ants were infected from the secondary cadaver, indicating an onward chain of transmission. Transmission via secondary cadavers could only happen after ants that died from the first exposure had fungal growth and the subsequent conidia production. Before 100% of ants within the nest were dead, *B*. *bassiana* growth from secondary cadavers was observed in 14 out of the 16 (88%) OCC nests and in 9 out of the 13 (69%) TCC nests. The remaining two and four nests, respectively, had *B*. *bassiana* growing from secondary cadavers before the end of the experiment, but after all the ants were dead (Fig 3). In the TOC nests, where all the cadavers were removed from the plaster nest (by the ants) to the empty arena, *B*. *bassiana* did not grow from the secondary cadavers within the 28 days of experiment. The plastic cage is less humid and less conducive to growth, compared to the plaster nests that mimic a natural nest setting in soil.

Based on the time of first death and the first observed fungal growth from secondary cadavers, we estimated the minimal number of ants directly infected by the introduced infectious cadaver (Fig 3 and S3 Table). Although there is some variation between the time of first death and fungal growth, we found that, on average, 62% of the ants (423 individuals) died before *B*. *bassiana* could grow from secondary cadavers (Fig 3), implying they were infected by the introduced infectious cadaver. An additional 22% of the ants (150 individuals) died after the fungus started growing from secondary cadavers, but before the minimal time lapsed between contact and death (based on the time of the first death after introduced cadaver on the beginning of the experiment) (Fig 3). This means that approximately 84% of the dead ants were killed following contact with the single infectious cadaver, experimentally introduced within the nest. The remaining 16% of the ants (109 individuals) could have been infected either by the introduced infectious cadavers or, potentially, by secondary cadavers. However, it is important to note that, in all the TOC nests (n = 6), 4 TCC nests and 2 OCC nests, 100% of ants were dead before *B*. *bassiana* growth was observed from secondary cadavers. Additionally, in 7 nests that had fungus growing from the secondary cadavers (out of 23), 100% of the ants died before the minimal time required for the fungus growing from the secondary cadaver to kill them. Taken together, for 19 of the nests exposed to *B*. *bassiana* (out of 35), 100% of the ants were infected by the introduced infectious cadaver

## Discussion

Despite the growing number of experimental studies on the behavioral mechanisms of social immunity in ant societies, little is known about how effective such responses are at the colony level, and if social immunity can, in fact, prevent disease progression within the nest environment. Here, we report the collapse of 100% of the tested sub-colonies following the introduction of a single infectious cadaver killed by *B*. *bassiana*, a generalist entomopathogenic fungus. We observed the progression of the fungus within the ant nest by not removing the secondary cadavers from the experiment (i.e. deaths that resulted from the 1^st^ inoculum, in this system, via an infectious cadaver). In our experiment, we recovered the fungus from 584 (86%) of the exposed ants. The confirmed infection per sub-colony ranged from 50 to 100% of the ants. This implies that a single introduced infectious cadaver is able to transmit within the nest, and social immunity did not prevent the collapse of the small sub-colonies under the conditions that we tested here.

As part of our assay, in 29 of the sub-colonies, the ants were restricted to the nest interior (16 one closed chamber (OCC) and 13 two connected closed chambers (TCC)). Closing the chamber prevents the ants from expressing behaviors such as social isolation and cadaver removal, which are considered important for the prevention of diseases spread within the colony [13]. Different from what we expected, the ability to leave the nest in the two connected open chamber (TOC) nests did not improve the survival of ants when compared to sub-colonies where the nests were closed and so the ants were prevented from expressing those behaviors (OCC and TTC). Neither did the opportunity to spatial segregate within the nest (in TCC) reduce the transmission; as similar levels of ants died across different nest architectures (Fig 1). Thus, we have no evidence that collective behavior related to occupation of space can benefit the colony in the presence of a single infectious cadaver.

It is important to consider that the size of our sub-colonies and the design of our nests may not represent mature colonies. One reason we chose small sizes is that they likely represent the effective group size of the small chambers a typical mature colony is divided into [44]. We also argue that the size of our sub-colonies and the design of our nests could represent the earlier stages of colony life. Young colonies, composed of queens and only few workers, are assumed to be more vulnerable to parasites [4], and this stage is suggested to be a critical bottleneck in the life cycle of ants [19]. As the number of workers increases, they presumably need to dig more tunnels and chambers, and could encounter infectious sporulating cadavers, likely of other insects. It could possibly result in the infections of the small colony, as we report here. In this context, recognizing the threat before exposure of all the workers within the chamber would be important. It is of course possible that the results could be different in a more complex architecture, such multiple chambered nests as found in mature ant colonies [44]. Whether our experimental set up represents either nascent colonies or modules of larger colony, or both, it is evident that collapse is possible when a single infectious cadaver is introduced. Another important note to make is that an infectious cadaver with *B*. *bassiana* can release a massive amount of spores [35, 36], contaminating the entire chamber and overwhelming the individual defenses of each organism and the collective defenses of the group. So far, studies on collective defenses of ants have focused on infected ants prior to death and, consequently, have not investigated the infectious sporulating stage of a cadaver. It is possible, of course, that the collective behavior can slow down, or perhaps even stop, the sporulation of a nestmate killed by the fungus. However, as this is a generalist parasite, infectious cadavers of other insects, on the surface or within the top layers of the soil, are also real threats. Thus, the transmission chain could be initiated due the encounter with other organisms and is not limited to the infectious cadavers of nestmates.

We found that the ants we tested appeared to quickly recognize the infectious cadavers and treat them shortly after their introduction in the nest. They were observed bending their gaster towards the infectious cadaver, likely spraying venom, which has anti-microbial properties [45, 46]. On the other hand, the ants seem to behave indifferently toward the control cadavers introduced into the chamber. It has been reported that ants are able to recognize infected food, infected environments and even infected nestmates [18, 28, 29, 33]. However, prior to our work, it was not clear how they would deal with infectious cadavers. Thus, although our observations were not quantified, they were in line with the previous literature on social immunity [18, 25, 41, 42]. The behavior reported here was not enough to spare the small colony from collapse, but a detailed study of how the ants behave towards infectious cadavers would be of interest.

Collective behavior of ants is seen as an important component of colony defense against generalized parasitic infections, but it is often studied in an individual context. Overall, studies show that particular mechanisms can improve the survival of focal treated ants [25, 45], but they rarely last for more than 14 days. This is a relatively short period of time and it is presumably justified because most ants in the experiments die due to infection. However, since the lifespan of a society is greater than its individual members it would seem important to measure the effect of disease for longer periods. Jaccoud et al. [32] observed the complete decline of colonies after 30 days of experiment, although towards the end, very few ants were confirmed to be infected. In the present studies, even ants that survived the 28 days of experiment were confirmed for *B*. *bassiana* infection post-mortem. Thus, the fungal infection started by the introduced infectious cadaver and progressed in a way that killed all the ants. We hope this study will encourage more research on the long-term effect of collective immune behavior and stronger links between social immunity and colony survival.

## Supporting Information

S1 Fig *Beauveria bassiana* growth from ant cadavers. The cadavers were kept inside the plaster chambers for 60 hours without live ants. These chambers had the same conditions as the chambers used in the experiments described here.
(PDF)

S2 Fig Nest architecture used in this study. (A) One closed chamber (OCC). (B) Two connected closed chambers (TCC). (C) Two connected open chambers (OTC). All the nests were made of plaster. The grey cylinders represent the tubes that were inserted into the chambers to feed the ants. The red dot shows the point where the infectious or control cadavers were introduced. In (B) and (C), the dashed line represents the blockage that limited the ants to the food chamber during the 48 hours of acclimation.
(PDF)

S3 Fig Before and after photographs of infectious cadavers introduced into the sub-colonies. The cadavers were left within the live ants for 24 hours.
(PDF)

S4 Fig Spatial segregation between live and dead ants within the chamber and pile of secondary cadavers organized by the ants (arrows). (A) One closed chamber. (B) Two closed chambers (TCC). Note that, in the TCC, the live ants occupied the secondary chamber while the cadavers where piled up in the food chamber.
(PDF)

S5 Fig Survival probability of ants exposed to infectious cadavers (dashed lines) or control cadaver (solid lines). The ants were housed in nests with distinct spatial configuration: one closed chamber (OCC, black lines), two closed chambers (TCC, grey lines) or two open chambers (TOC, red lines). The survival of the ants depended on the type of cadaver they were exposed (infectious or control). The nest design did not affect the survival of the ants. There was no interaction between nest design and cadaver type.
(PDF)

S1 Table Source colony and number of individuals used in each sub-colony. (PDF)

S2 Table Survival data collected during 28 days of experiment. The data was used to perform the ratio hazard analysis.
(PDF)

S3 Table Data used to estimate the minimal number of ants directly infected by the introduced infectious cadaver. (PDF)
