# Supplementary material for: Disease in the Society: Infectious Cadavers Result in Collapse of Ant Sub-Colonies
Source: PLoS One. 2016 Aug 16;11(8):e0160820. doi: 10.1371/journal.pone.0160820 (PMC4986943; doi:10.1371/journal.pone.0160820)

Figure S1: *Beauveria bassiana* growth from cadavers kept inside the plaster chambers for 60 hours without live ants. These chambers had the same conditions as the nests used in the experiments.

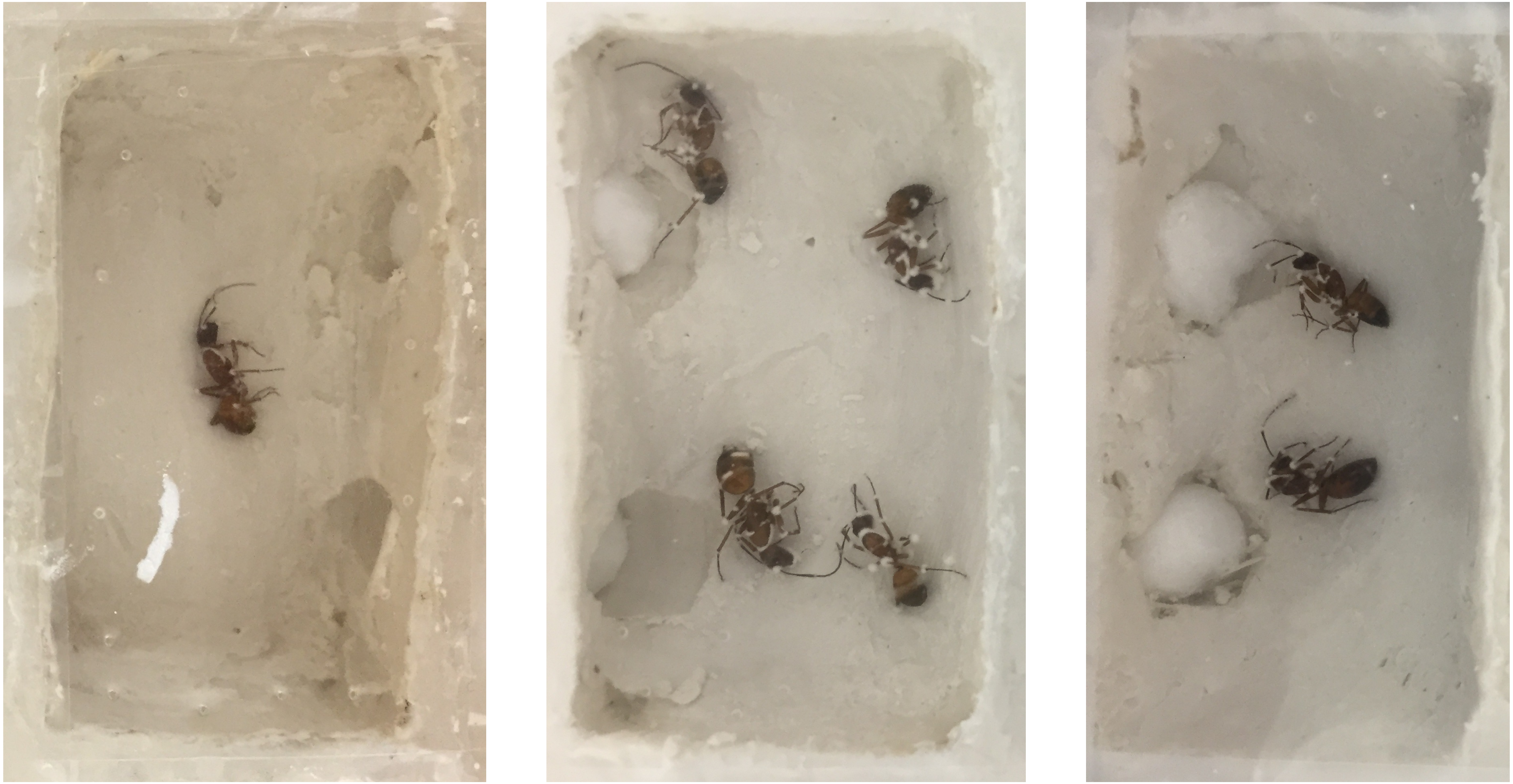

Supplement: S1 Fig — The cadavers were kept inside the plaster chambers for 60 hours without live ants. These chambers had the same conditions as the chambers used in the experiments described here. (PDF) [file pone.0160820.s001.pdf]
