# Supplementary material for: Disease in the Society: Infectious Cadavers Result in Collapse of Ant Sub-Colonies
Source: PLoS One. 2016 Aug 16;11(8):e0160820. doi: 10.1371/journal.pone.0160820 (PMC4986943; doi:10.1371/journal.pone.0160820)

Figure S2: Plaster nests design used in this study. (A) One closed chamber (OCC). (B) Two connected closed chambers (TCC). (C) Two conected open chambers (OTC). The grey cilinders reperesent the tubes that were inseted into the chambers to feed the ants. The red dot shows where the infectious or control cadaver was introduced. In (B) and (C), the dashed line represents the blockage that limited the ants to the food chamber during the 48 hours of acclimation.

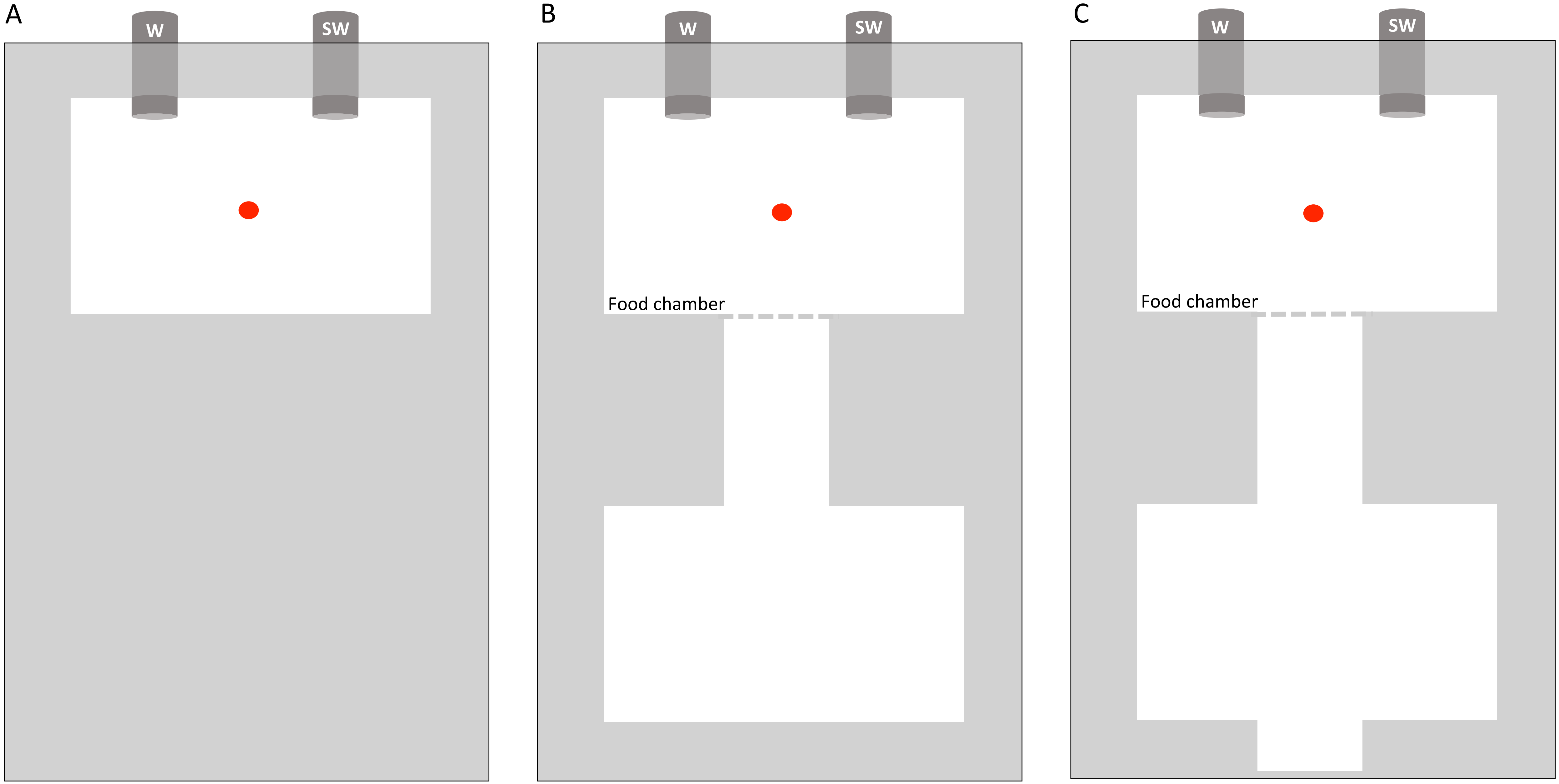

Supplement: S2 Fig — (A) One closed chamber (OCC). (B) Two connected closed chambers (TCC). (C) Two connected open chambers (OTC). All the nests were made of plaster. The grey cylinders represent the tubes that were inserted into the chambers to feed the ants. The red dot shows the point where the infectious or control cadavers were introduced. In (B) and (C), the dashed line represents the blockage that limited the ants to the food chamber during the 48 hours of acclimation. (PDF) [file pone.0160820.s002.pdf]
