# Supplementary material for: Disease in the Society: Infectious Cadavers Result in Collapse of Ant Sub-Colonies
Source: PLoS One. 2016 Aug 16;11(8):e0160820. doi: 10.1371/journal.pone.0160820 (PMC4986943; doi:10.1371/journal.pone.0160820)

Figure S3: Before and after photographs of infectious cadavers introduced into the sub-colonies. The cadavers were left within the ants for 24 hours.

<u>One Closed Chamber</u>

Flem 3 OCC

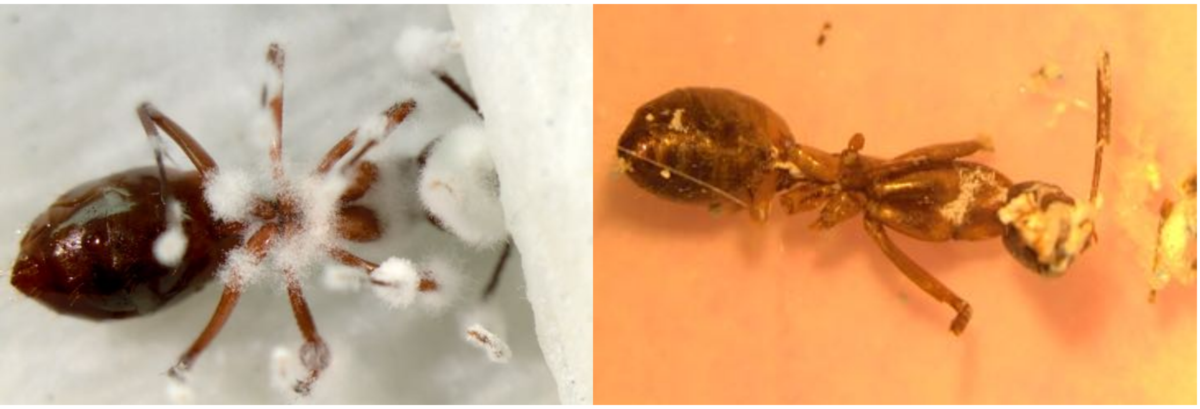

Flem 6 OCC

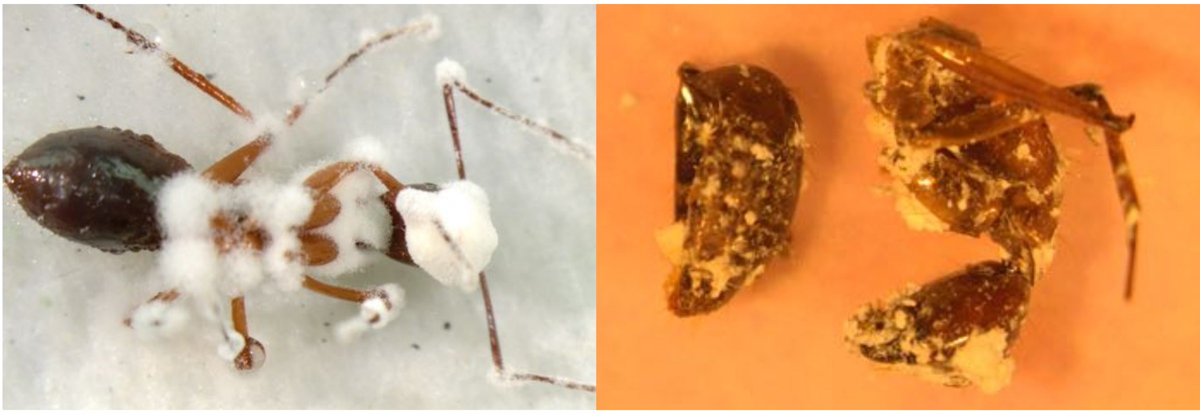

Flem 8 OCC

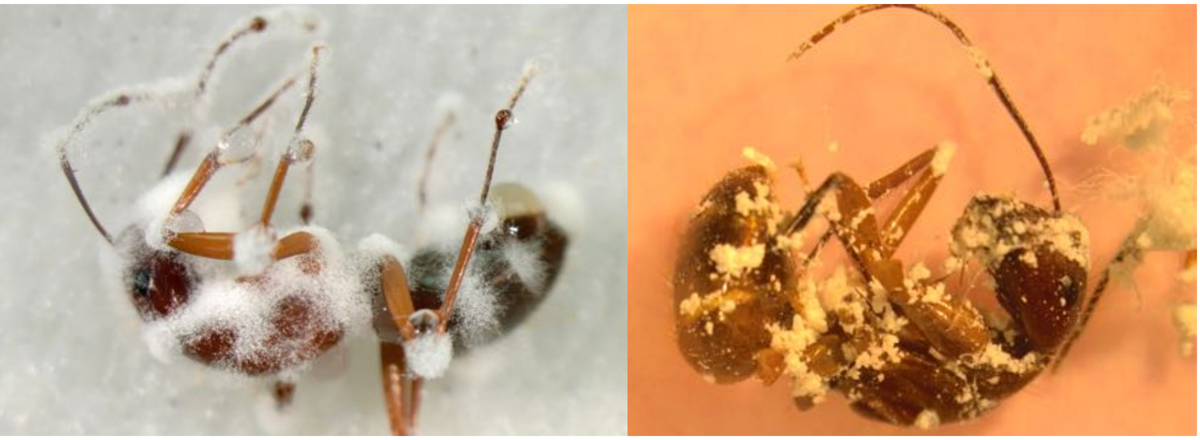

Flem 11 OCC

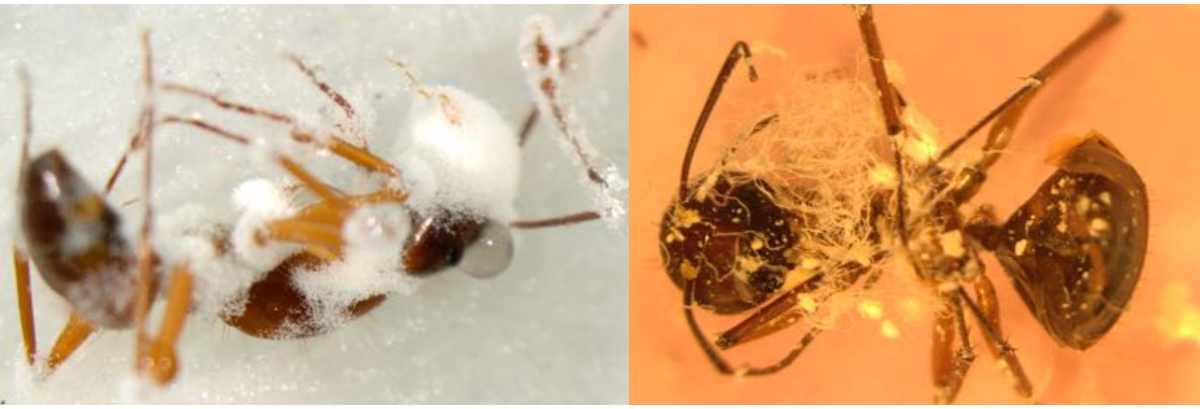

KFM 1 OCC

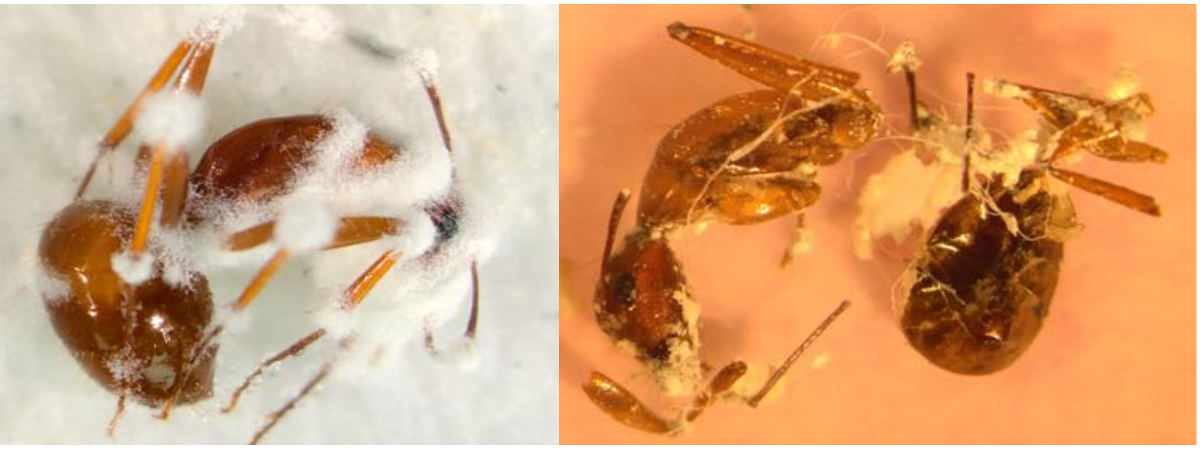

KFM 3 OCC

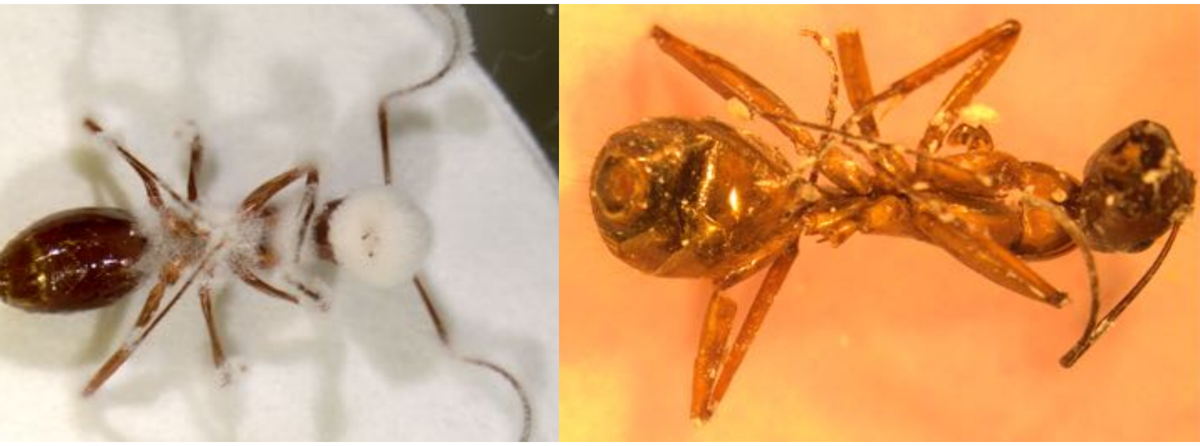

KFM 11 OCC

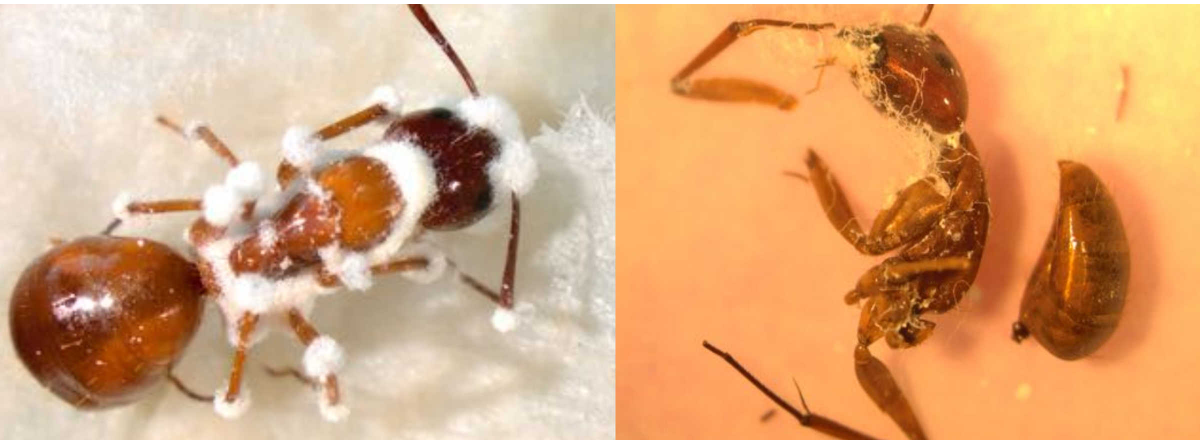

<u>Two Closed Chambers</u>

Flem 5 TCC

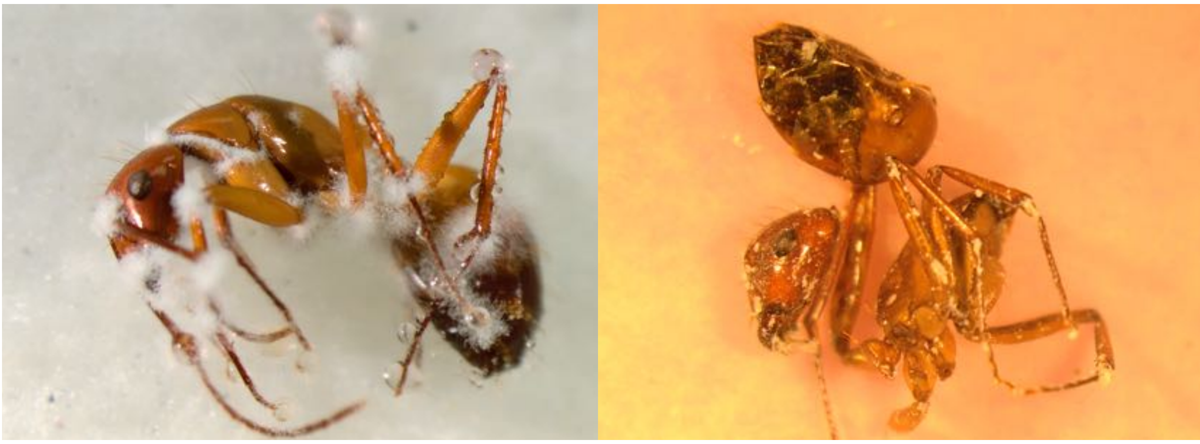

Flem 7 TCC

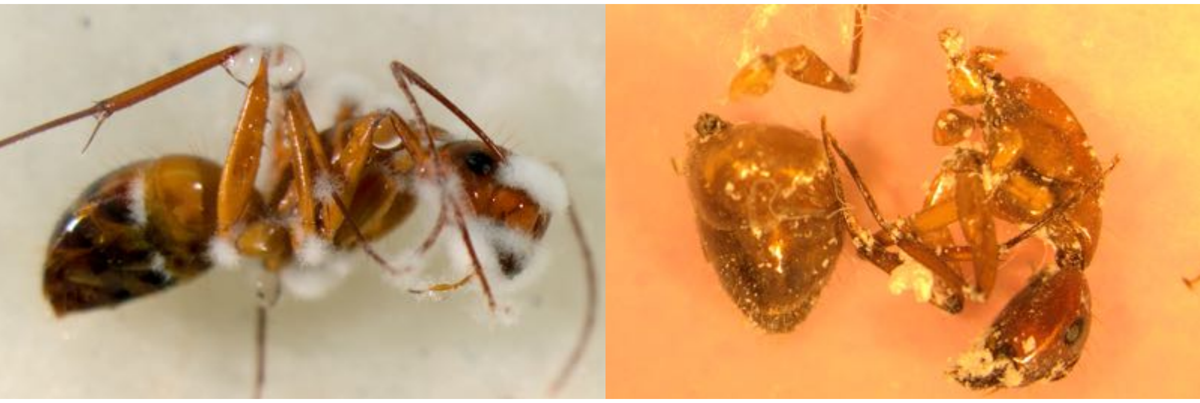

Flem 11 TCC

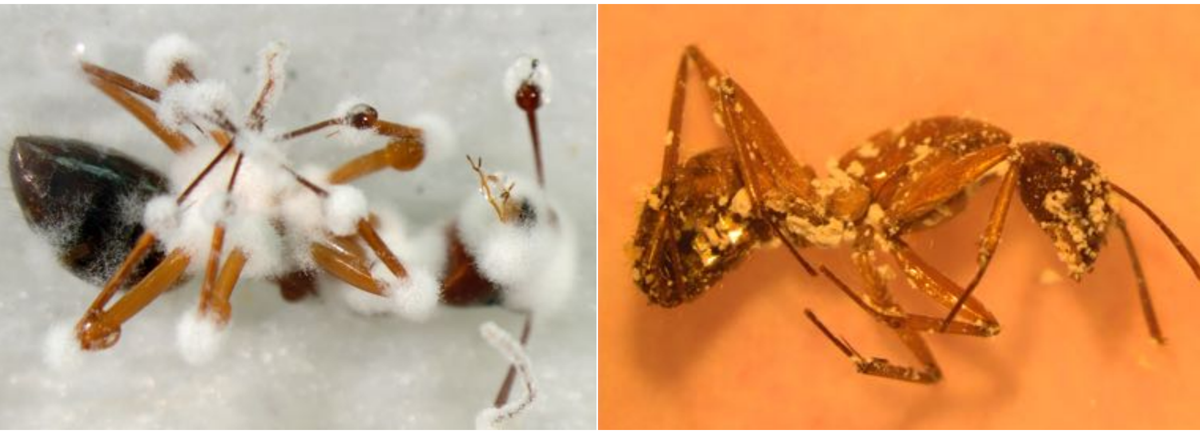

KFM 16 TCC

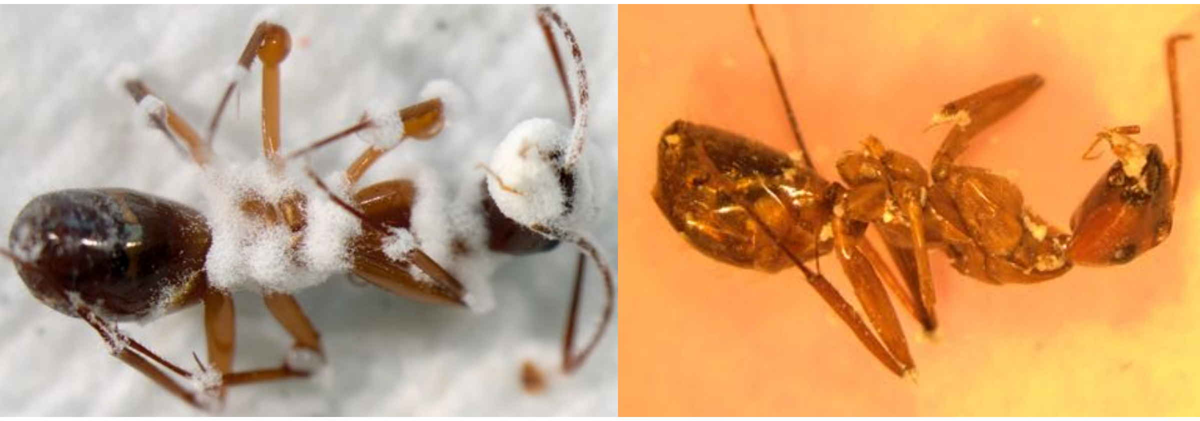

KFM 26 TCC

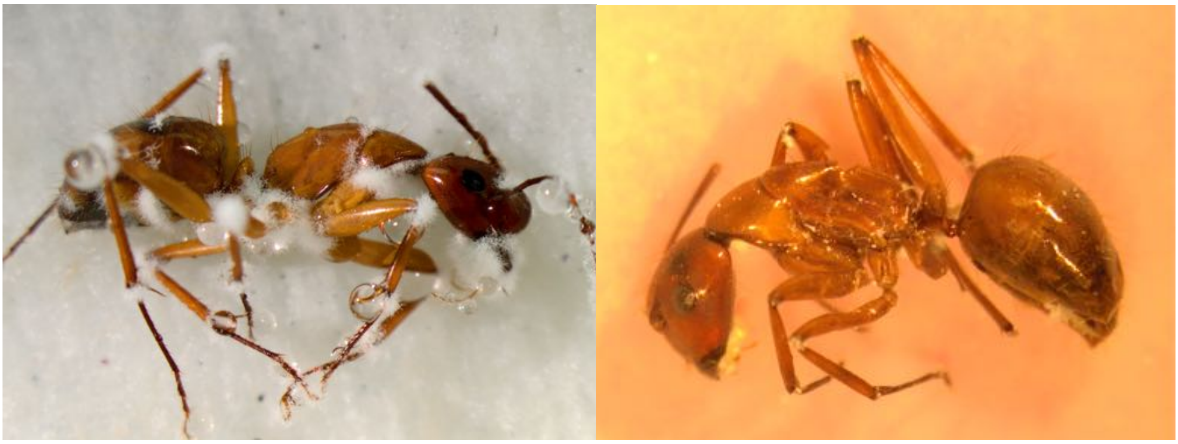

<u>Two Open Chambers</u>

KFM 1 TOC

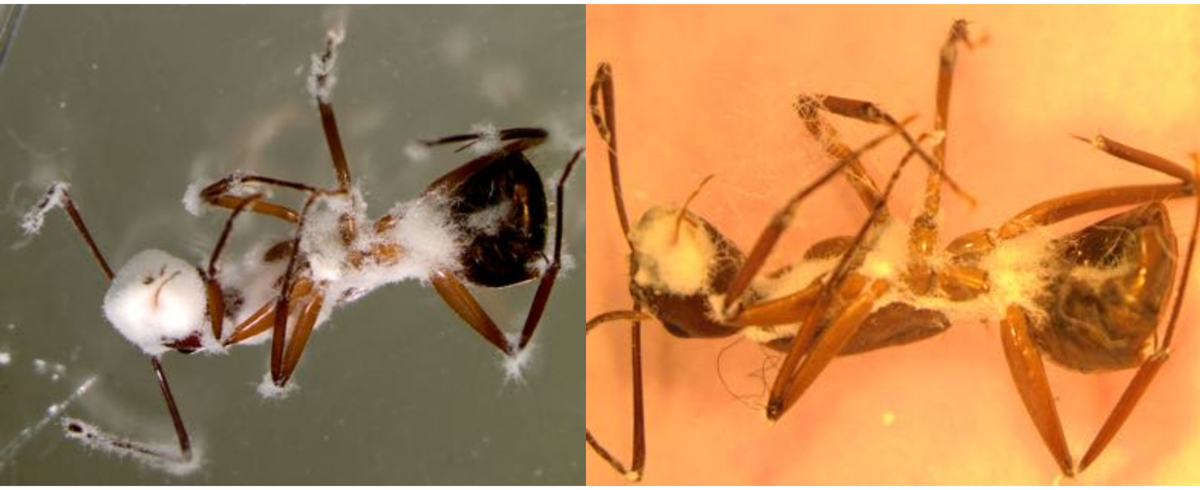

KMF 3 TOC

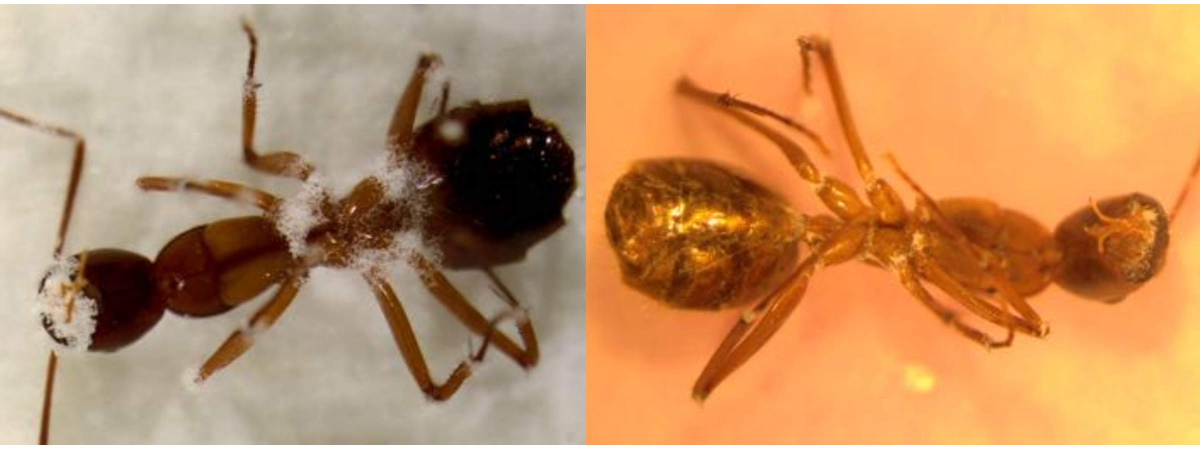

KMF 7 TOC

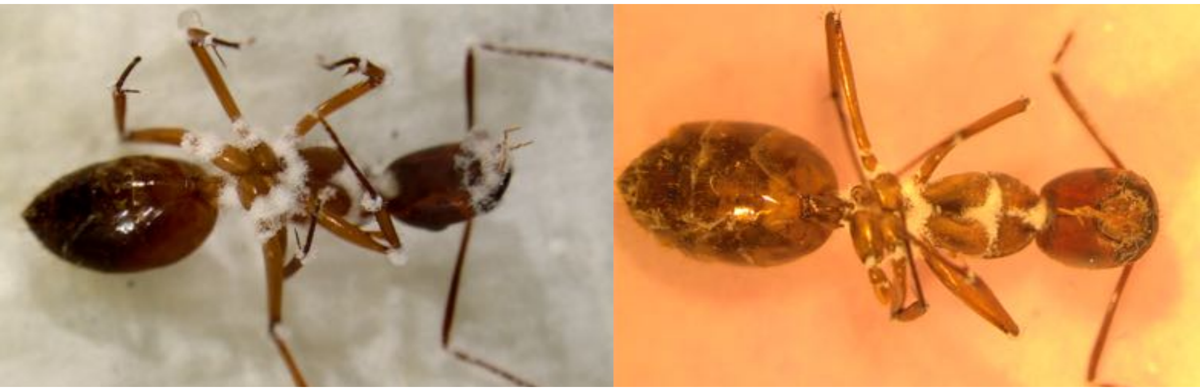

Flem7 TOC

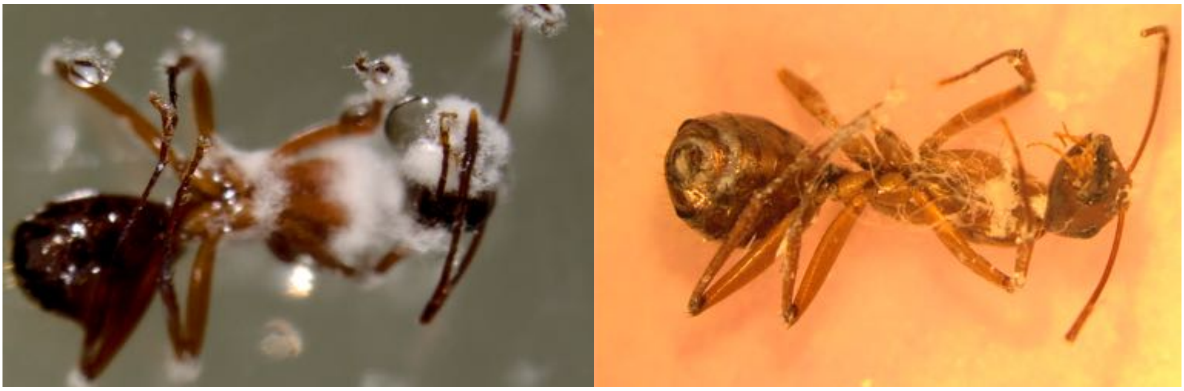

Supplement: S3 Fig — The cadavers were left within the live ants for 24 hours. (PDF) [file pone.0160820.s003.pdf]
