# Supplementary material for: Disease in the Society: Infectious Cadavers Result in Collapse of Ant Sub-Colonies
Source: PLoS One. 2016 Aug 16;11(8):e0160820. doi: 10.1371/journal.pone.0160820 (PMC4986943; doi:10.1371/journal.pone.0160820)

Figure S4: Spatial segregation between live and dead ants within the chamber. And piled of secondary cadavers (arrows) (A) One closed chamber. (B) Two closed chambers (TCC). Note that in the TCC the live ants occupy the secondary chamber while the cadavers where piled up in the food chamber.

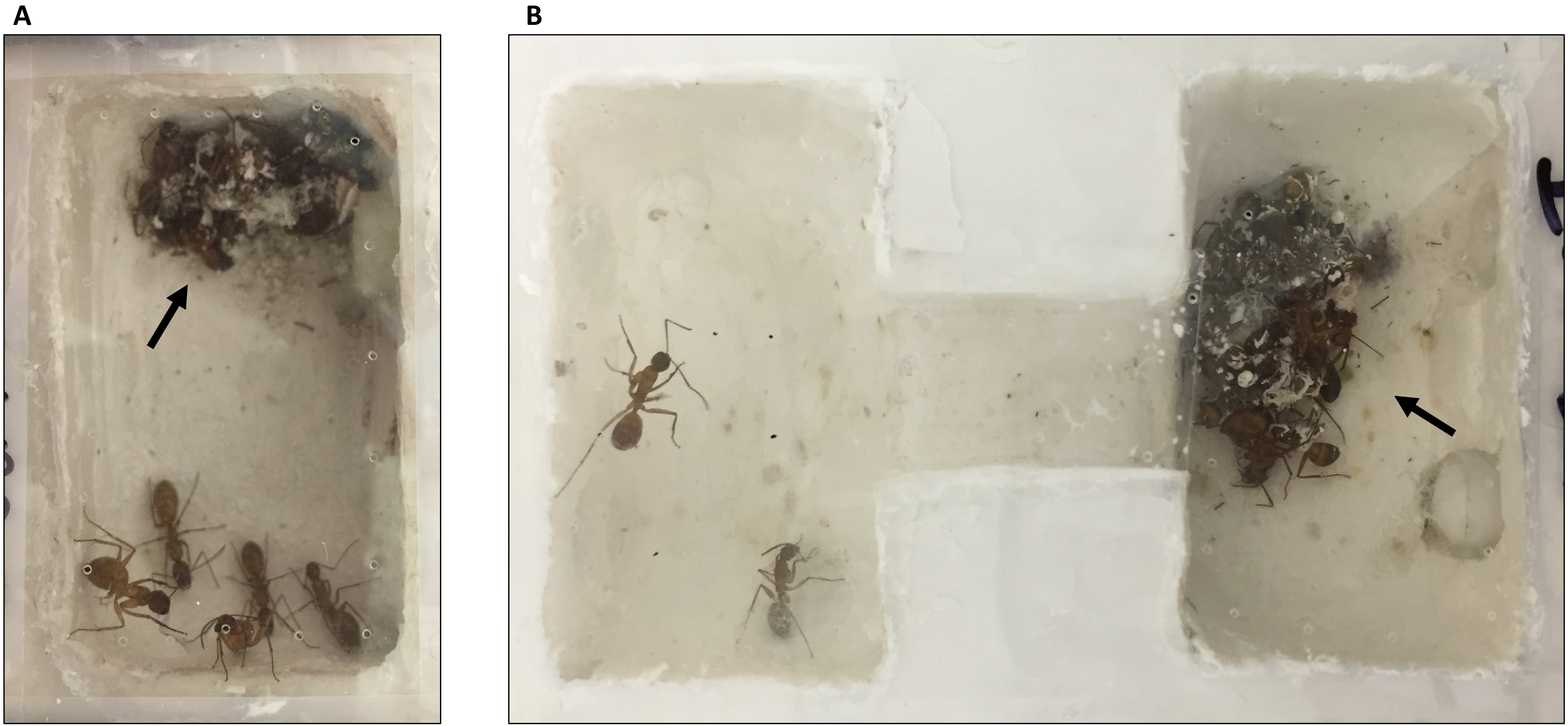

Supplement: S4 Fig — (A) One closed chamber. (B) Two closed chambers (TCC). Note that, in the TCC, the live ants occupied the secondary chamber while the cadavers where piled up in the food chamber. (PDF) [file pone.0160820.s004.pdf]
