# Supplementary material for: Disease in the Society: Infectious Cadavers Result in Collapse of Ant Sub-Colonies
Source: PLoS One. 2016 Aug 16;11(8):e0160820. doi: 10.1371/journal.pone.0160820 (PMC4986943; doi:10.1371/journal.pone.0160820)

Figure S5: Survival probability of ants exposed to infectous cadavers (dashed lines) or control cadaver (solid lines). The ants were housed in distinct nests design: one closed chamber (OCC, black lines), two closed chambers (TCC, grey lines) or two open chambers (TOC, red lines). The survival of the ants decreased depending on the type of cadaver they were exposed (infectious or control), but the nest design did not affect the survival of the ants. There was no interaction between nest design and cadaver type.

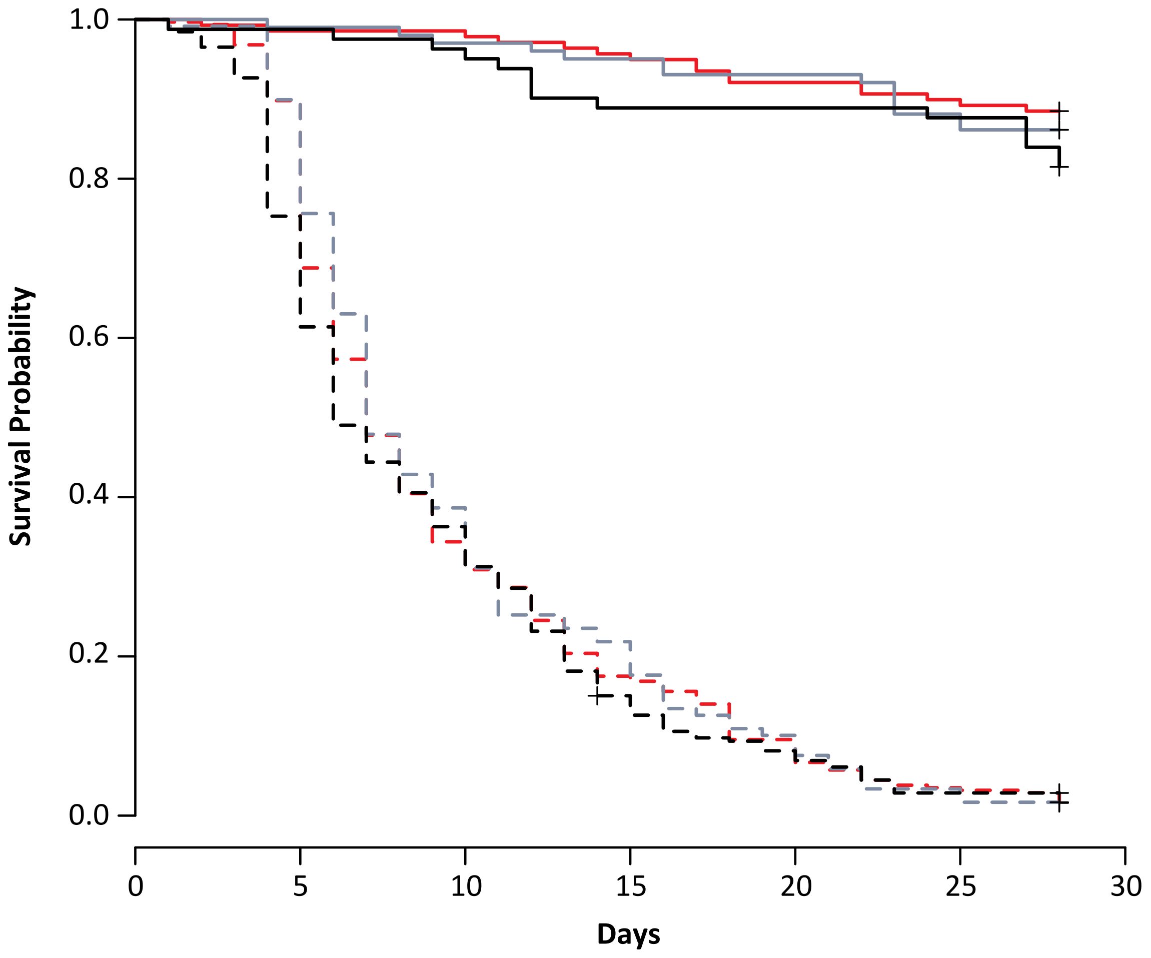

Supplement: S5 Fig — The ants were housed in nests with distinct spatial configuration: one closed chamber (OCC, black lines), two closed chambers (TCC, grey lines) or two open chambers (TOC, red lines). The survival of the ants depended on the type of cadaver they were exposed (infectious or control). The nest design did not affect the survival of the ants. There was no interaction between nest design and cadaver type. (PDF) [file pone.0160820.s005.pdf]
