# Supplementary material for: Disease in the Society: Infectious Cadavers Result in Collapse of Ant Sub-Colonies
Source: PLoS One. 2016 Aug 16;11(8):e0160820. doi: 10.1371/journal.pone.0160820 (PMC4986943; doi:10.1371/journal.pone.0160820)
Supplement: S1 Table — (PDF) [file pone.0160820.s006.pdf]

Table S1: Source colony and number of individuals used in each sub-colony

	Infectious cadaver			Control cadaver		
Colony	OCC	TCC	TOC	OCC	TCC	TOC
Flem11	20	22	-	-	20	-
Flem3	21	20	-	-	-	-
Flem5	20	20	-	-	-	-
Flem6	20	18	-	-	20	-
Flem7	21	20	21	-	-	-
Flem8	20	20	-	-	20	-
KFM1	20	20	20	19	20	21
KFM11	20	20	-	20	-	-
KFM13	20	16	-	19	-	-
KFM16	21	21	-	21	-	-
KFM21	21	-	-	-	-	-
KFM22	20	20	21	20	-	21
KFM26	19	22	20	20	-	22
KFM3	20	-	20	-	-	19
KFM4	20	20	-	20	-	-
KFM7	-	-	17	-	-	16
