# Supplementary material for: Disease in the Society: Infectious Cadavers Result in Collapse of Ant Sub-Colonies
Source: PLoS One. 2016 Aug 16;11(8):e0160820. doi: 10.1371/journal.pone.0160820 (PMC4986943; doi:10.1371/journal.pone.0160820)
Supplement: S2 Table — The data was used to perform the ratio hazard analysis. (PDF) [file pone.0160820.s007.pdf]

Table S2: Survival data collected during 28 days of experiment. The data was used to perform the Cox proportional-hazard analysis.

ant	colony	chamber	treatment	days	sensor	growth
1	KFM26	Two	Cadaver	9	1	Day 12
2	KFM26	Two	Cadaver	10	1	
3	KFM26	Two	Cadaver	11	1	
4	KFM26	Two	Cadaver	11	1	
5	KFM26	Two	Cadaver	11	1	
6	KFM26	Two	Cadaver	12	1	
7	KFM26	Two	Cadaver	12	1	
8	KFM26	Two	Cadaver	12	1	
9	KFM26	Two	Cadaver	12	1	
10	KFM26	Two	Cadaver	12	1	
11	KFM26	Two	Cadaver	12	1	
12	KFM26	Two	Cadaver	13	1	
13	KFM26	Two	Cadaver	13	1	
14	KFM26	Two	Cadaver	14	1	
15	KFM26	Two	Cadaver	14	1	
16	KFM26	Two	Cadaver	14	1	
17	KFM26	Two	Cadaver	15	1	
18	KFM26	Two	Cadaver	15	1	
19	KFM26	Two	Cadaver	16	1	
20	KFM26	Two	Cadaver	19	1	
21	KFM26	Two	Cadaver	19	1	
22	KFM26	Two	Cadaver	22	1	
23	KFM11	Two	Cadaver	9	1	na
24	KFM11	Two	Cadaver	11	1	
25	KFM11	Two	Cadaver	20	1	
26	KFM11	Two	Cadaver	20	1	
27	KFM11	Two	Cadaver	20	1	
28	KFM11	Two	Cadaver	21	1	
29	KFM11	Two	Cadaver	21	1	
30	KFM11	Two	Cadaver	22	1	
31	KFM11	Two	Cadaver	22	1	
32	KFM11	Two	Cadaver	22	1	
33	KFM11	Two	Cadaver	23	1	
34	KFM11	Two	Cadaver	23	1	
35	KFM11	Two	Cadaver	23	1	
36	KFM11	Two	Cadaver	23	1	
37	KFM11	Two	Cadaver	28	0	
38	KFM11	Two	Cadaver	28	0	
39	KFM11	Two	Cadaver	28	0	

40	KFM11	Two	Cadaver	28	0	
41	KFM11	Two	Cadaver	28	0	
42	KFM11	Two	Cadaver	28	0	
43	Flem11	Two	Cadaver	4	1	Day 6
44	Flem11	Two	Cadaver	4	1	
45	Flem11	Two	Cadaver	4	1	
46	Flem11	Two	Cadaver	4	1	
47	Flem11	Two	Cadaver	4	1	
48	Flem11	Two	Cadaver	4	1	
49	Flem11	Two	Cadaver	4	1	
50	Flem11	Two	Cadaver	4	1	
51	Flem11	Two	Cadaver	4	1	
52	Flem11	Two	Cadaver	5	1	
53	Flem11	Two	Cadaver	5	1	
54	Flem11	Two	Cadaver	5	1	
55	Flem11	Two	Cadaver	5	1	
56	Flem11	Two	Cadaver	5	1	
57	Flem11	Two	Cadaver	5	1	
58	Flem11	Two	Cadaver	5	1	
59	Flem11	Two	Cadaver	6	1	
60	Flem11	Two	Cadaver	6	1	
61	Flem11	Two	Cadaver	7	1	
62	Flem11	Two	Cadaver	7	1	
63	Flem11	Two	Cadaver	7	1	
64	Flem11	Two	Cadaver	7	1	
65	Flem3	One	Cadaver	4	1	Day 7
66	Flem3	One	Cadaver	4	1	
67	Flem3	One	Cadaver	4	1	
68	Flem3	One	Cadaver	4	1	
69	Flem3	One	Cadaver	4	1	
70	Flem3	One	Cadaver	5	1	
71	Flem3	One	Cadaver	5	1	
72	Flem3	One	Cadaver	5	1	
73	Flem3	One	Cadaver	5	1	
74	Flem3	One	Cadaver	6	1	
75	Flem3	One	Cadaver	6	1	
76	Flem3	One	Cadaver	6	1	
77	Flem3	One	Cadaver	6	1	
78	Flem3	One	Cadaver	6	1	
79	Flem3	One	Cadaver	7	1	
80	Flem3	One	Cadaver	7	1	
81	Flem3	One	Cadaver	8	1	

82	Flem3	One	Cadaver	8	1	
83	Flem3	One	Cadaver	8	1	
84	Flem3	One	Cadaver	8	1	
85	Flem3	One	Cadaver	8	1	
86	Flem8	One	Cadaver	4	1	Day 7
87	Flem8	One	Cadaver	4	1	
88	Flem8	One	Cadaver	5	1	
89	Flem8	One	Cadaver	5	1	
90	Flem8	One	Cadaver	5	1	
91	Flem8	One	Cadaver	5	1	
92	Flem8	One	Cadaver	5	1	
93	Flem8	One	Cadaver	6	1	
94	Flem8	One	Cadaver	6	1	
95	Flem8	One	Cadaver	6	1	
96	Flem8	One	Cadaver	7	1	
97	Flem8	One	Cadaver	7	1	
98	Flem8	One	Cadaver	7	1	
99	Flem8	One	Cadaver	7	1	
100	Flem8	One	Cadaver	7	1	
101	Flem8	One	Cadaver	8	1	
102	Flem8	One	Cadaver	8	1	
103	Flem8	One	Cadaver	8	1	
104	Flem8	One	Cadaver	9	1	
105	Flem8	One	Cadaver	9	1	
106	Flem3	Two	Cadaver	1	1	Day 7
107	Flem3	Two	Cadaver	4	1	
108	Flem3	Two	Cadaver	4	1	
109	Flem3	Two	Cadaver	5	1	
110	Flem3	Two	Cadaver	5	1	
111	Flem3	Two	Cadaver	5	1	
112	Flem3	Two	Cadaver	6	1	
113	Flem3	Two	Cadaver	6	1	
114	Flem3	Two	Cadaver	7	1	
115	Flem3	Two	Cadaver	8	1	
116	Flem3	Two	Cadaver	9	1	
117	Flem3	Two	Cadaver	11	1	
118	Flem3	Two	Cadaver	12	1	
119	Flem3	Two	Cadaver	12	1	
120	Flem3	Two	Cadaver	13	1	
121	Flem3	Two	Cadaver	13	1	
122	Flem3	Two	Cadaver	13	1	
123	Flem3	Two	Cadaver	14	1	

124	Flem3	Two	Cadaver	15	1	
125	Flem3	Two	Cadaver	28	0	
126	KFM16	One	Cadaver	4	1	Day 8
127	KFM16	One	Cadaver	4	1	
128	KFM16	One	Cadaver	5	1	
129	KFM16	One	Cadaver	5	1	
130	KFM16	One	Cadaver	5	1	
131	KFM16	One	Cadaver	5	1	
132	KFM16	One	Cadaver	5	1	
133	KFM16	One	Cadaver	6	1	
134	KFM16	One	Cadaver	8	1	
135	KFM16	One	Cadaver	8	1	
136	KFM16	One	Cadaver	8	1	
137	KFM16	One	Cadaver	9	1	
138	KFM16	One	Cadaver	9	1	
139	KFM16	One	Cadaver	9	1	
140	KFM16	One	Cadaver	9	1	
141	KFM16	One	Cadaver	10	1	
142	KFM16	One	Cadaver	12	1	
143	KFM16	One	Cadaver	14	1	
144	KFM16	One	Cadaver	14	1	
145	KFM16	One	Cadaver	14	1	
146	KFM16	One	Cadaver	14	1	
147	Flem8	Two	Cadaver	4	1	na
148	Flem8	Two	Cadaver	4	1	
149	Flem8	Two	Cadaver	4	1	
150	Flem8	Two	Cadaver	4	1	
151	Flem8	Two	Cadaver	5	1	
152	Flem8	Two	Cadaver	5	1	
153	Flem8	Two	Cadaver	5	1	
154	Flem8	Two	Cadaver	5	1	
155	Flem8	Two	Cadaver	5	1	
156	Flem8	Two	Cadaver	5	1	
157	Flem8	Two	Cadaver	5	1	
158	Flem8	Two	Cadaver	5	1	
159	Flem8	Two	Cadaver	5	1	
160	Flem8	Two	Cadaver	6	1	
161	Flem8	Two	Cadaver	6	1	
162	Flem8	Two	Cadaver	6	1	
163	Flem8	Two	Cadaver	6	1	
164	Flem8	Two	Cadaver	6	1	
165	Flem8	Two	Cadaver	6	1	

166	Flem8	Two	Cadaver	10	1	
167	Flem7	Two	Cadaver	1	1	Day 7
168	Flem7	Two	Cadaver	2	1	
169	Flem7	Two	Cadaver	4	1	
170	Flem7	Two	Cadaver	4	1	
171	Flem7	Two	Cadaver	4	1	
172	Flem7	Two	Cadaver	5	1	
173	Flem7	Two	Cadaver	5	1	
174	Flem7	Two	Cadaver	5	1	
175	Flem7	Two	Cadaver	5	1	
176	Flem7	Two	Cadaver	6	1	
177	Flem7	Two	Cadaver	6	1	
178	Flem7	Two	Cadaver	6	1	
179	Flem7	Two	Cadaver	7	1	
180	Flem7	Two	Cadaver	8	1	
181	Flem7	Two	Cadaver	8	1	
182	Flem7	Two	Cadaver	8	1	
183	Flem7	Two	Cadaver	9	1	
184	Flem7	Two	Cadaver	9	1	
185	Flem7	Two	Cadaver	9	1	
186	Flem7	Two	Cadaver	13	1	
187	KFM16	Two	Cadaver	3	1	Day 6
188	KFM16	Two	Cadaver	3	1	
189	KFM16	Two	Cadaver	3	1	
190	KFM16	Two	Cadaver	4	1	
191	KFM16	Two	Cadaver	4	1	
192	KFM16	Two	Cadaver	4	1	
193	KFM16	Two	Cadaver	4	1	
194	KFM16	Two	Cadaver	6	1	
195	KFM16	Two	Cadaver	6	1	
196	KFM16	Two	Cadaver	6	1	
197	KFM16	Two	Cadaver	6	1	
198	KFM16	Two	Cadaver	6	1	
199	KFM16	Two	Cadaver	6	1	
200	KFM16	Two	Cadaver	7	1	
201	KFM16	Two	Cadaver	7	1	
202	KFM16	Two	Cadaver	8	1	
203	KFM16	Two	Cadaver	9	1	
204	KFM16	Two	Cadaver	10	1	
205	KFM16	Two	Cadaver	10	1	
206	KFM16	Two	Cadaver	11	1	
207	KFM16	Two	Cadaver	11	1	

208	KFM1	Two	Cadaver	2	1	Day 8
209	KFM1	Two	Cadaver	2	1	
210	KFM1	Two	Cadaver	3	1	
211	KFM1	Two	Cadaver	3	1	
212	KFM1	Two	Cadaver	3	1	
213	KFM1	Two	Cadaver	3	1	
214	KFM1	Two	Cadaver	4	1	
215	KFM1	Two	Cadaver	4	1	
216	KFM1	Two	Cadaver	4	1	
217	KFM1	Two	Cadaver	4	1	
218	KFM1	Two	Cadaver	6	1	
219	KFM1	Two	Cadaver	6	1	
220	KFM1	Two	Cadaver	6	1	
221	KFM1	Two	Cadaver	6	1	
222	KFM1	Two	Cadaver	7	1	
223	KFM1	Two	Cadaver	8	1	
224	KFM1	Two	Cadaver	10	1	
225	KFM1	Two	Cadaver	13	1	
226	KFM1	Two	Cadaver	13	1	
227	KFM1	Two	Cadaver	14	1	
228	KFM26	One	Cadaver	5	1	Day 9
229	KFM26	One	Cadaver	5	1	
230	KFM26	One	Cadaver	5	1	
231	KFM26	One	Cadaver	5	1	
232	KFM26	One	Cadaver	5	1	
233	KFM26	One	Cadaver	6	1	
234	KFM26	One	Cadaver	7	1	
235	KFM26	One	Cadaver	7	1	
236	KFM26	One	Cadaver	7	1	
237	KFM26	One	Cadaver	7	1	
238	KFM26	One	Cadaver	9	1	
239	KFM26	One	Cadaver	9	1	
240	KFM26	One	Cadaver	11	1	
241	KFM26	One	Cadaver	13	1	
242	KFM26	One	Cadaver	13	1	
243	KFM26	One	Cadaver	14	1	
244	KFM26	One	Cadaver	18	1	
245	KFM26	One	Cadaver	18	1	
246	KFM26	One	Cadaver	18	1	
247	KFM13	Two	Cadaver	2	1	na
248	KFM13	Two	Cadaver	4	1	
249	KFM13	Two	Cadaver	4	1	

250	KFM13	Two	Cadaver	4	1	
251	KFM13	Two	Cadaver	4	1	
252	KFM13	Two	Cadaver	4	1	
253	KFM13	Two	Cadaver	5	1	
254	KFM13	Two	Cadaver	5	1	
255	KFM13	Two	Cadaver	5	1	
256	KFM13	Two	Cadaver	5	1	
257	KFM13	Two	Cadaver	5	1	
258	KFM13	Two	Cadaver	5	1	
259	KFM13	Two	Cadaver	6	1	
260	KFM13	Two	Cadaver	6	1	
261	KFM13	Two	Cadaver	6	1	
262	KFM13	Two	Cadaver	7	1	
263	KFM13	One	Cadaver	3	1	Day 10
264	KFM13	One	Cadaver	5	1	
265	KFM13	One	Cadaver	5	1	
266	KFM13	One	Cadaver	5	1	
267	KFM13	One	Cadaver	5	1	
268	KFM13	One	Cadaver	5	1	
269	KFM13	One	Cadaver	6	1	
270	KFM13	One	Cadaver	6	1	
271	KFM13	One	Cadaver	6	1	
272	KFM13	One	Cadaver	7	1	
273	KFM13	One	Cadaver	8	1	
274	KFM13	One	Cadaver	8	1	
275	KFM13	One	Cadaver	12	1	
276	KFM13	One	Cadaver	13	1	
277	KFM13	One	Cadaver	16	1	
278	KFM13	One	Cadaver	16	1	
279	KFM13	One	Cadaver	16	1	
280	KFM13	One	Cadaver	16	1	
281	KFM13	One	Cadaver	17	1	
282	KFM13	One	Cadaver	18	1	
283	KFM22	One	Cadaver	4	1	Day 7
284	KFM22	One	Cadaver	4	1	
285	KFM22	One	Cadaver	5	1	
286	KFM22	One	Cadaver	5	1	
287	KFM22	One	Cadaver	5	1	
288	KFM22	One	Cadaver	5	1	
289	KFM22	One	Cadaver	5	1	
290	KFM22	One	Cadaver	5	1	
291	KFM22	One	Cadaver	5	1	

292	KFM22	One	Cadaver	5	1	
293	KFM22	One	Cadaver	5	1	
294	KFM22	One	Cadaver	5	1	
295	KFM22	One	Cadaver	6	1	
296	KFM22	One	Cadaver	6	1	
297	KFM22	One	Cadaver	8	1	
298	KFM22	One	Cadaver	10	1	
299	KFM22	One	Cadaver	12	1	
300	KFM22	One	Cadaver	13	1	
301	KFM22	One	Cadaver	13	1	
302	KFM22	One	Cadaver	14	1	
303	KFM1	One	Cadaver	3	1	Day 9
304	KFM1	One	Cadaver	4	1	
305	KFM1	One	Cadaver	5	1	
306	KFM1	One	Cadaver	5	1	
307	KFM1	One	Cadaver	5	1	
308	KFM1	One	Cadaver	5	1	
309	KFM1	One	Cadaver	5	1	
310	KFM1	One	Cadaver	7	1	
311	KFM1	One	Cadaver	7	1	
312	KFM1	One	Cadaver	8	1	
313	KFM1	One	Cadaver	8	1	
314	KFM1	One	Cadaver	9	1	
315	KFM1	One	Cadaver	10	1	
316	KFM1	One	Cadaver	13	1	
317	KFM1	One	Cadaver	13	1	
318	KFM1	One	Cadaver	18	1	
319	KFM1	One	Cadaver	18	1	
320	KFM1	One	Cadaver	20	1	
321	KFM1	One	Cadaver	22	1	
322	KFM1	One	Cadaver	24	1	
323	KFM22	Two	Cadaver	1	1	
324	KFM22	Two	Cadaver	1	1	na
325	KFM22	Two	Cadaver	3	1	
326	KFM22	Two	Cadaver	4	1	
327	KFM22	Two	Cadaver	4	1	
328	KFM22	Two	Cadaver	4	1	
329	KFM22	Two	Cadaver	4	1	
330	KFM22	Two	Cadaver	4	1	
331	KFM22	Two	Cadaver	5	1	
332	KFM22	Two	Cadaver	5	1	
333	KFM22	Two	Cadaver	6	1	

334	KFM22	Two	Cadaver	6	1	
335	KFM22	Two	Cadaver	8	1	
336	KFM22	Two	Cadaver	9	1	
337	KFM22	Two	Cadaver	9	1	
338	KFM22	Two	Cadaver	10	1	
339	KFM22	Two	Cadaver	12	1	
340	KFM22	Two	Cadaver	14	1	
341	KFM22	Two	Cadaver	16	1	
342	KFM22	Two	Cadaver	18	1	
343	KFM11	One	Cadaver	4	1	Day 7
344	KFM11	One	Cadaver	4	1	
345	KFM11	One	Cadaver	5	1	
346	KFM11	One	Cadaver	5	1	
347	KFM11	One	Cadaver	5	1	
348	KFM11	One	Cadaver	6	1	
349	KFM11	One	Cadaver	6	1	
350	KFM11	One	Cadaver	6	1	
351	KFM11	One	Cadaver	6	1	
352	KFM11	One	Cadaver	6	1	
353	KFM11	One	Cadaver	7	1	
354	KFM11	One	Cadaver	7	1	
355	KFM11	One	Cadaver	9	1	
356	KFM11	One	Cadaver	10	1	
357	KFM11	One	Cadaver	12	1	
358	KFM11	One	Cadaver	13	1	
359	KFM11	One	Cadaver	13	1	
360	KFM11	One	Cadaver	13	1	
361	KFM11	One	Cadaver	15	1	
362	KFM11	One	Cadaver	15	1	
363	Flem6	One	Cadaver	3	1	Day 7
364	Flem6	One	Cadaver	3	1	
365	Flem6	One	Cadaver	4	1	
366	Flem6	One	Cadaver	4	1	
367	Flem6	One	Cadaver	4	1	
368	Flem6	One	Cadaver	4	1	
369	Flem6	One	Cadaver	5	1	
370	Flem6	One	Cadaver	5	1	
371	Flem6	One	Cadaver	5	1	
372	Flem6	One	Cadaver	5	1	
373	Flem6	One	Cadaver	5	1	
374	Flem6	One	Cadaver	5	1	
375	Flem6	One	Cadaver	5	1	

376	Flem6	One	Cadaver	5	1	
377	Flem6	One	Cadaver	5	1	
378	Flem6	One	Cadaver	5	1	
379	Flem6	One	Cadaver	6	1	
380	Flem6	One	Cadaver	7	1	
381	Flem6	One	Cadaver	7	1	
382	Flem6	One	Cadaver	8	1	
383	Flem5	One	Cadaver	3	1	Day 7
384	Flem5	One	Cadaver	3	1	
385	Flem5	One	Cadaver	3	1	
386	Flem5	One	Cadaver	4	1	
387	Flem5	One	Cadaver	5	1	
388	Flem5	One	Cadaver	5	1	
389	Flem5	One	Cadaver	5	1	
390	Flem5	One	Cadaver	5	1	
391	Flem5	One	Cadaver	6	1	
392	Flem5	One	Cadaver	6	1	
393	Flem5	One	Cadaver	6	1	
394	Flem5	One	Cadaver	6	1	
395	Flem5	One	Cadaver	6	1	
396	Flem5	One	Cadaver	7	1	
397	Flem5	One	Cadaver	8	1	
398	Flem5	One	Cadaver	9	1	
399	Flem5	One	Cadaver	9	1	
400	Flem5	One	Cadaver	12	1	
401	Flem5	One	Cadaver	12	1	
402	Flem5	One	Cadaver	13	1	
403	KFM21	One	Cadaver	2	1	Day 6
404	KFM21	One	Cadaver	5	1	
405	KFM21	One	Cadaver	5	1	
406	KFM21	One	Cadaver	6	1	
407	KFM21	One	Cadaver	7	1	
408	KFM21	One	Cadaver	7	1	
409	KFM21	One	Cadaver	7	1	
410	KFM21	One	Cadaver	10	1	
411	KFM21	One	Cadaver	10	1	
412	KFM21	One	Cadaver	12	1	
413	KFM21	One	Cadaver	12	1	
414	KFM21	One	Cadaver	18	1	
415	KFM21	One	Cadaver	18	1	
416	KFM21	One	Cadaver	18	1	
417	KFM21	One	Cadaver	18	1	

418	KFM21	One	Cadaver	18	1	
419	KFM21	One	Cadaver	20	1	
420	KFM21	One	Cadaver	20	1	
421	KFM21	One	Cadaver	22	1	
422	KFM21	One	Cadaver	22	1	
423	KFM21	One	Cadaver	22	1	
424	Flem5	Two	Cadaver	4	1	Day 9
425	Flem5	Two	Cadaver	5	1	
426	Flem5	Two	Cadaver	6	1	
427	Flem5	Two	Cadaver	6	1	
428	Flem5	Two	Cadaver	7	1	
429	Flem5	Two	Cadaver	7	1	
430	Flem5	Two	Cadaver	8	1	
431	Flem5	Two	Cadaver	8	1	
432	Flem5	Two	Cadaver	9	1	
433	Flem5	Two	Cadaver	10	1	
434	Flem5	Two	Cadaver	10	1	
435	Flem5	Two	Cadaver	10	1	
436	Flem5	Two	Cadaver	12	1	
437	Flem5	Two	Cadaver	12	1	
438	Flem5	Two	Cadaver	12	1	
439	Flem5	Two	Cadaver	12	1	
440	Flem5	Two	Cadaver	13	1	
441	Flem5	Two	Cadaver	13	1	
442	Flem5	Two	Cadaver	13	1	
443	Flem5	Two	Cadaver	14	1	
444	KFM04	Two	Cadaver	4	1	Day 10
445	KFM04	Two	Cadaver	5	1	
446	KFM04	Two	Cadaver	6	1	
447	KFM04	Two	Cadaver	8	1	
448	KFM04	Two	Cadaver	9	1	
449	KFM04	Two	Cadaver	10	1	
450	KFM04	Two	Cadaver	10	1	
451	KFM04	Two	Cadaver	10	1	
452	KFM04	Two	Cadaver	12	1	
453	KFM04	Two	Cadaver	13	1	
454	KFM04	Two	Cadaver	14	1	
455	KFM04	Two	Cadaver	15	1	
456	KFM04	Two	Cadaver	15	1	
457	KFM04	Two	Cadaver	15	1	
458	KFM04	Two	Cadaver	16	1	
459	KFM04	Two	Cadaver	16	1	

460	KFM04	Two	Cadaver	16	1	
461	KFM04	Two	Cadaver	17	1	
462	KFM04	Two	Cadaver	17	1	
463	KFM04	Two	Cadaver	19	1	
464	Flem6	Two	Cadaver	2	1	Day 8
465	Flem6	Two	Cadaver	3	1	
466	Flem6	Two	Cadaver	3	1	
467	Flem6	Two	Cadaver	4	1	
468	Flem6	Two	Cadaver	4	1	
469	Flem6	Two	Cadaver	4	1	
470	Flem6	Two	Cadaver	4	1	
471	Flem6	Two	Cadaver	4	1	
472	Flem6	Two	Cadaver	4	1	
473	Flem6	Two	Cadaver	4	1	
474	Flem6	Two	Cadaver	5	1	
475	Flem6	Two	Cadaver	5	1	
476	Flem6	Two	Cadaver	5	1	
477	Flem6	Two	Cadaver	6	1	
478	Flem6	Two	Cadaver	10	1	
479	Flem6	Two	Cadaver	13	1	
480	Flem6	Two	Cadaver	14	0	
481	Flem6	Two	Cadaver	14	0	
482	Flem11	One	Cadaver	3	1	Day 7
483	Flem11	One	Cadaver	5	1	
484	Flem11	One	Cadaver	5	1	
485	Flem11	One	Cadaver	5	1	
486	Flem11	One	Cadaver	5	1	
487	Flem11	One	Cadaver	6	1	
488	Flem11	One	Cadaver	6	1	
489	Flem11	One	Cadaver	6	1	
490	Flem11	One	Cadaver	6	1	
491	Flem11	One	Cadaver	6	1	
492	Flem11	One	Cadaver	7	1	
493	Flem11	One	Cadaver	7	1	
494	Flem11	One	Cadaver	7	1	
495	Flem11	One	Cadaver	7	1	
496	Flem11	One	Cadaver	8	1	
497	Flem11	One	Cadaver	8	1	
498	Flem11	One	Cadaver	9	1	
499	Flem11	One	Cadaver	10	1	
500	Flem11	One	Cadaver	12	1	
501	Flem11	One	Cadaver	13	1	

502	KFM04	One	Cadaver	1	1	Day 12
503	KFM04	One	Cadaver	5	1	
504	KFM04	One	Cadaver	7	1	
505	KFM04	One	Cadaver	8	1	
506	KFM04	One	Cadaver	8	1	
507	KFM04	One	Cadaver	10	1	
508	KFM04	One	Cadaver	21	1	
509	KFM04	One	Cadaver	23	1	
510	KFM04	One	Cadaver	23	1	
511	KFM04	One	Cadaver	25	1	
512	KFM04	One	Cadaver	27	1	
513	KFM04	One	Cadaver	28	1	
514	KFM04	One	Cadaver	30	1	
515	KFM04	One	Cadaver	33	1	
516	KFM04	One	Cadaver	33	1	
517	KFM04	One	Cadaver	34	0	
518	KFM04	One	Cadaver	34	0	
519	KFM04	One	Cadaver	34	0	
520	KFM04	One	Cadaver	34	0	
521	KFM04	One	Cadaver	34	0	
522	Flem7	One	Cadaver	9	1	Day 17
523	Flem7	One	Cadaver	9	1	
524	Flem7	One	Cadaver	10	1	
525	Flem7	One	Cadaver	12	1	
526	Flem7	One	Cadaver	14	1	
527	Flem7	One	Cadaver	14	1	
528	Flem7	One	Cadaver	17	1	
529	Flem7	One	Cadaver	17	1	
530	Flem7	One	Cadaver	17	1	
531	Flem7	One	Cadaver	17	1	
532	Flem7	One	Cadaver	18	1	
533	Flem7	One	Cadaver	18	1	
534	Flem7	One	Cadaver	18	1	
535	Flem7	One	Cadaver	20	1	
536	Flem7	One	Cadaver	20	1	
537	Flem7	One	Cadaver	20	1	
538	Flem7	One	Cadaver	20	1	
539	Flem7	One	Cadaver	20	1	
540	Flem7	One	Cadaver	20	1	
541	Flem7	One	Cadaver	21	1	
542	Flem7	One	Cadaver	21	1	
543	Flem7	Open	Cadaver	4	1	na

544	Flem7	Open	Cadaver	4	1	
545	Flem7	Open	Cadaver	5	1	
546	Flem7	Open	Cadaver	5	1	
547	Flem7	Open	Cadaver	5	1	
548	Flem7	Open	Cadaver	5	1	
549	Flem7	Open	Cadaver	5	1	
550	Flem7	Open	Cadaver	5	1	
551	Flem7	Open	Cadaver	5	1	
552	Flem7	Open	Cadaver	6	1	
553	Flem7	Open	Cadaver	7	1	
554	Flem7	Open	Cadaver	7	1	
555	Flem7	Open	Cadaver	7	1	
556	Flem7	Open	Cadaver	7	1	
557	Flem7	Open	Cadaver	8	1	
558	Flem7	Open	Cadaver	8	1	
559	Flem7	Open	Cadaver	9	1	
560	Flem7	Open	Cadaver	10	1	
561	Flem7	Open	Cadaver	11	1	
562	Flem7	Open	Cadaver	13	1	
563	Flem7	Open	Cadaver	16	1	
564	KFM22	Open	Cadaver	4	1	na
565	KFM22	Open	Cadaver	5	1	
566	KFM22	Open	Cadaver	5	1	
567	KFM22	Open	Cadaver	6	1	
568	KFM22	Open	Cadaver	6	1	
569	KFM22	Open	Cadaver	6	1	
570	KFM22	Open	Cadaver	6	1	
571	KFM22	Open	Cadaver	8	1	
572	KFM22	Open	Cadaver	9	1	
573	KFM22	Open	Cadaver	9	1	
574	KFM22	Open	Cadaver	10	1	
575	KFM22	Open	Cadaver	11	1	
576	KFM22	Open	Cadaver	11	1	
577	KFM22	Open	Cadaver	14	1	
578	KFM22	Open	Cadaver	15	1	
579	KFM22	Open	Cadaver	15	1	
580	KFM22	Open	Cadaver	16	1	
581	KFM22	Open	Cadaver	16	1	
582	KFM22	Open	Cadaver	17	1	
583	KFM22	Open	Cadaver	18	1	
584	KFM22	Open	Cadaver	20	1	
585	KFM1	Open	Cadaver	4	1	na

586	KFM1	Open	Cadaver	5	1	
587	KFM1	Open	Cadaver	6	1	
588	KFM1	Open	Cadaver	7	1	
589	KFM1	Open	Cadaver	7	1	
590	KFM1	Open	Cadaver	7	1	
591	KFM1	Open	Cadaver	8	1	
592	KFM1	Open	Cadaver	10	1	
593	KFM1	Open	Cadaver	11	1	
594	KFM1	Open	Cadaver	15	1	
595	KFM1	Open	Cadaver	20	1	
596	KFM1	Open	Cadaver	20	1	
597	KFM1	Open	Cadaver	21	1	
598	KFM1	Open	Cadaver	21	1	
599	KFM1	Open	Cadaver	22	1	
600	KFM1	Open	Cadaver	22	1	
601	KFM1	Open	Cadaver	22	1	
602	KFM1	Open	Cadaver	25	1	
603	KFM1	Open	Cadaver	28	0	
604	KFM1	Open	Cadaver	28	0	
605	KFM26	Open	Cadaver	4	1	na
606	KFM26	Open	Cadaver	4	1	
607	KFM26	Open	Cadaver	5	1	
608	KFM26	Open	Cadaver	5	1	
609	KFM26	Open	Cadaver	5	1	
610	KFM26	Open	Cadaver	6	1	
611	KFM26	Open	Cadaver	6	1	
612	KFM26	Open	Cadaver	6	1	
613	KFM26	Open	Cadaver	6	1	
614	KFM26	Open	Cadaver	7	1	
615	KFM26	Open	Cadaver	7	1	
616	KFM26	Open	Cadaver	7	1	
617	KFM26	Open	Cadaver	7	1	
618	KFM26	Open	Cadaver	9	1	
619	KFM26	Open	Cadaver	10	1	
620	KFM26	Open	Cadaver	10	1	
621	KFM26	Open	Cadaver	10	1	
622	KFM26	Open	Cadaver	10	1	
623	KFM26	Open	Cadaver	11	1	
624	KFM26	Open	Cadaver	16	1	
625	KFM3	One	Cadaver	4	1	na
626	KFM3	One	Cadaver	5	1	
627	KFM3	One	Cadaver	6	1	

628	KFM3	One	Cadaver	6	1	
629	KFM3	One	Cadaver	6	1	
630	KFM3	One	Cadaver	7	1	
631	KFM3	One	Cadaver	7	1	
632	KFM3	One	Cadaver	7	1	
633	KFM3	One	Cadaver	9	1	
634	KFM3	One	Cadaver	9	1	
635	KFM3	One	Cadaver	10	1	
636	KFM3	One	Cadaver	10	1	
637	KFM3	One	Cadaver	11	1	
638	KFM3	One	Cadaver	11	1	
639	KFM3	One	Cadaver	11	1	
640	KFM3	One	Cadaver	11	1	
641	KFM3	One	Cadaver	11	1	
642	KFM3	One	Cadaver	12	1	
643	KFM3	One	Cadaver	13	1	
644	KFM3	One	Cadaver	14	1	
645	KFM7	Open	Cadaver	1	1	na
646	KFM7	Open	Cadaver	4	1	
647	KFM7	Open	Cadaver	4	1	
648	KFM7	Open	Cadaver	6	1	
649	KFM7	Open	Cadaver	6	1	
650	KFM7	Open	Cadaver	6	1	
651	KFM7	Open	Cadaver	7	1	
652	KFM7	Open	Cadaver	7	1	
653	KFM7	Open	Cadaver	7	1	
654	KFM7	Open	Cadaver	8	1	
655	KFM7	Open	Cadaver	9	1	
656	KFM7	Open	Cadaver	10	1	
657	KFM7	Open	Cadaver	11	1	
658	KFM7	Open	Cadaver	13	1	
659	KFM7	Open	Cadaver	15	1	
660	KFM7	Open	Cadaver	19	1	
661	KFM7	Open	Cadaver	25	1	
662	KFM3	Open	Cadaver	4	1	na
663	KFM3	Open	Cadaver	4	1	
664	KFM3	Open	Cadaver	4	1	
665	KFM3	Open	Cadaver	5	1	
666	KFM3	Open	Cadaver	5	1	
667	KFM3	Open	Cadaver	5	1	
668	KFM3	Open	Cadaver	5	1	
669	KFM3	Open	Cadaver	6	1	

670	KFM3	Open	Cadaver	6	1	
671	KFM3	Open	Cadaver	7	1	
672	KFM3	Open	Cadaver	7	1	
673	KFM3	Open	Cadaver	7	1	
674	KFM3	Open	Cadaver	7	1	
675	KFM3	Open	Cadaver	8	1	
676	KFM3	Open	Cadaver	10	1	
677	KFM3	Open	Cadaver	11	1	
678	KFM3	Open	Cadaver	14	1	
679	KFM3	Open	Cadaver	15	1	
680	KFM3	Open	Cadaver	16	1	
681	KFM3	Open	Cadaver	18	1	
682	KFM1	One	Control	4	1	na
683	KFM1	One	Control	10	1	
684	KFM1	One	Control	11	1	
685	KFM1	One	Control	15	1	
686	KFM1	One	Control	18	1	
687	KFM1	One	Control	28	0	
688	KFM1	One	Control	28	0	
689	KFM1	One	Control	28	0	
690	KFM1	One	Control	28	0	
691	KFM1	One	Control	28	0	
692	KFM1	One	Control	28	0	
693	KFM1	One	Control	28	0	
694	KFM1	One	Control	28	0	
695	KFM1	One	Control	28	0	
696	KFM1	One	Control	28	0	
697	KFM1	One	Control	28	0	
698	KFM1	One	Control	28	0	
699	KFM1	One	Control	28	0	
700	KFM1	One	Control	28	0	
701	KFM16	One	Control	24	1	na
702	KFM16	One	Control	27	1	
703	KFM16	One	Control	28	0	
704	KFM16	One	Control	28	0	
705	KFM16	One	Control	28	0	
706	KFM16	One	Control	28	0	
707	KFM16	One	Control	28	0	
708	KFM16	One	Control	28	0	
709	KFM16	One	Control	28	0	
710	KFM16	One	Control	28	0	
711	KFM16	One	Control	28	0	

712	KFM16	One	Control	28	0	
713	KFM16	One	Control	28	0	
714	KFM16	One	Control	28	0	
715	KFM16	One	Control	28	0	
716	KFM16	One	Control	28	0	
717	KFM16	One	Control	28	0	
718	KFM16	One	Control	28	0	
719	KFM16	One	Control	28	0	
720	KFM16	One	Control	28	0	
721	KFM16	One	Control	28	0	
722	Flem11	Two	Control	11	1	na
723	Flem11	Two	Control	28	0	
724	Flem11	Two	Control	28	0	
725	Flem11	Two	Control	28	0	
726	Flem11	Two	Control	28	0	
727	Flem11	Two	Control	28	0	
728	Flem11	Two	Control	28	0	
729	Flem11	Two	Control	28	0	
730	Flem11	Two	Control	28	0	
731	Flem11	Two	Control	28	0	
732	Flem11	Two	Control	28	0	
733	Flem11	Two	Control	28	0	
734	Flem11	Two	Control	28	0	
735	Flem11	Two	Control	28	0	
736	Flem11	Two	Control	28	0	
737	Flem11	Two	Control	28	0	
738	Flem11	Two	Control	28	0	
739	Flem11	Two	Control	28	0	
740	Flem11	Two	Control	28	0	
741	Flem11	Two	Control	28	0	
742	Flem11	Two	Control	28	0	
743	Flem8	Two	Control	9	1	na
744	Flem8	Two	Control	27	1	
745	Flem8	Two	Control	27	1	
746	Flem8	Two	Control	27	1	
747	Flem8	Two	Control	28	1	
748	Flem8	Two	Control	28	1	
749	Flem8	Two	Control	28	0	
750	Flem8	Two	Control	28	0	
751	Flem8	Two	Control	28	0	
752	Flem8	Two	Control	28	0	
753	Flem8	Two	Control	28	0	

754	Flem8	Two	Control	28	0	
755	Flem8	Two	Control	28	0	
756	Flem8	Two	Control	28	0	
757	Flem8	Two	Control	28	0	
758	Flem8	Two	Control	28	0	
759	Flem8	Two	Control	28	0	
760	Flem8	Two	Control	28	0	
761	Flem8	Two	Control	28	0	
762	Flem8	Two	Control	28	0	
763	KFM1	Two	Control	1	1	na
764	KFM1	Two	Control	6	1	
765	KFM1	Two	Control	10	1	
766	KFM1	Two	Control	12	1	
767	KFM1	Two	Control	12	1	
768	KFM1	Two	Control	12	1	
769	KFM1	Two	Control	28	0	
770	KFM1	Two	Control	28	0	
771	KFM1	Two	Control	28	0	
772	KFM1	Two	Control	28	0	
773	KFM1	Two	Control	28	0	
774	KFM1	Two	Control	28	0	
775	KFM1	Two	Control	28	0	
776	KFM1	Two	Control	28	0	
777	KFM1	Two	Control	28	0	
778	KFM1	Two	Control	28	0	
779	KFM1	Two	Control	28	0	
780	KFM1	Two	Control	28	0	
781	KFM1	Two	Control	28	0	
782	KFM1	Two	Control	28	0	
783	Flem6	Two	Control	14	1	na
784	Flem6	Two	Control	24	1	
785	Flem6	Two	Control	28	0	
786	Flem6	Two	Control	28	0	
787	Flem6	Two	Control	28	0	
788	Flem6	Two	Control	28	0	
789	Flem6	Two	Control	28	0	
790	Flem6	Two	Control	28	0	
791	Flem6	Two	Control	28	0	
792	Flem6	Two	Control	28	0	
793	Flem6	Two	Control	28	0	
794	Flem6	Two	Control	28	0	
795	Flem6	Two	Control	28	0	

796	Flem6	Two	Control	28	0	
797	Flem6	Two	Control	28	0	
798	Flem6	Two	Control	28	0	
799	Flem6	Two	Control	28	0	
800	Flem6	Two	Control	28	0	
801	Flem6	Two	Control	28	0	
802	Flem6	Two	Control	28	0	
803	KFM11	One	Control	31	0	na
804	KFM11	One	Control	31	0	
805	KFM11	One	Control	31	0	
806	KFM11	One	Control	31	0	
807	KFM11	One	Control	31	0	
808	KFM11	One	Control	31	0	
809	KFM11	One	Control	31	0	
810	KFM11	One	Control	31	0	
811	KFM11	One	Control	31	0	
812	KFM11	One	Control	31	0	
813	KFM11	One	Control	31	0	
814	KFM11	One	Control	31	0	
815	KFM11	One	Control	31	0	
816	KFM11	One	Control	31	0	
817	KFM11	One	Control	31	0	
818	KFM11	One	Control	31	0	
819	KFM11	One	Control	31	0	
820	KFM11	One	Control	31	0	
821	KFM11	One	Control	31	0	
822	KFM11	One	Control	31	0	
823	KFM26	Open	Control	28	0	na
824	KFM26	Open	Control	28	0	
825	KFM26	Open	Control	28	0	
826	KFM26	Open	Control	28	0	
827	KFM26	Open	Control	28	0	
828	KFM26	Open	Control	28	0	
829	KFM26	Open	Control	28	0	
830	KFM26	Open	Control	28	0	
831	KFM26	Open	Control	28	0	
832	KFM26	Open	Control	28	0	
833	KFM26	Open	Control	28	0	
834	KFM26	Open	Control	28	0	
835	KFM26	Open	Control	28	0	
836	KFM26	Open	Control	28	0	
837	KFM26	Open	Control	28	0	

838	KFM26	Open	Control	28	0	
839	KFM26	Open	Control	28	0	
840	KFM26	Open	Control	28	0	
841	KFM26	Open	Control	28	0	
842	KFM26	Open	Control	28	0	
843	KFM26	Open	Control	28	0	
844	KFM26	Open	Control	28	0	
845	KFM22	Open	Control	9	1	na
846	KFM22	Open	Control	23	1	
847	KFM22	Open	Control	25	1	
848	KFM22	Open	Control	28	0	
849	KFM22	Open	Control	28	0	
850	KFM22	Open	Control	28	0	
851	KFM22	Open	Control	28	0	
852	KFM22	Open	Control	28	0	
853	KFM22	Open	Control	28	0	
854	KFM22	Open	Control	28	0	
855	KFM22	Open	Control	28	0	
856	KFM22	Open	Control	28	0	
857	KFM22	Open	Control	28	0	
858	KFM22	Open	Control	28	0	
859	KFM22	Open	Control	28	0	
860	KFM22	Open	Control	28	0	
861	KFM22	Open	Control	28	0	
862	KFM22	Open	Control	28	0	
863	KFM22	Open	Control	28	0	
864	KFM22	Open	Control	28	0	
865	KFM22	Open	Control	28	0	
866	KFM3	Open	Control	16	1	na
867	KFM3	Open	Control	22	1	
868	KFM3	Open	Control	23	1	
869	KFM3	Open	Control	28	0	
870	KFM3	Open	Control	28	0	
871	KFM3	Open	Control	28	0	
872	KFM3	Open	Control	28	0	
873	KFM3	Open	Control	28	0	
874	KFM3	Open	Control	28	0	
875	KFM3	Open	Control	28	0	
876	KFM3	Open	Control	28	0	
877	KFM3	Open	Control	28	0	
878	KFM3	Open	Control	28	0	
879	KFM3	Open	Control	28	0	

880	KFM3	Open	Control	28	0	
881	KFM3	Open	Control	28	0	
882	KFM3	Open	Control	28	0	
883	KFM3	Open	Control	28	0	
884	KFM3	Open	Control	28	0	
885	KFM7	Open	Control	4	1	na
886	KFM7	Open	Control	12	1	
887	KFM7	Open	Control	13	1	
888	KFM7	Open	Control	16	1	
889	KFM7	Open	Control	23	1	
890	KFM7	Open	Control	28	0	
891	KFM7	Open	Control	28	0	
892	KFM7	Open	Control	28	0	
893	KFM7	Open	Control	28	0	
894	KFM7	Open	Control	28	0	
895	KFM7	Open	Control	28	0	
896	KFM7	Open	Control	28	0	
897	KFM7	Open	Control	28	0	
898	KFM7	Open	Control	28	0	
899	KFM7	Open	Control	28	0	
900	KFM7	Open	Control	28	0	
901	KFM7	Open	Control	28	0	
902	KFM7	Open	Control	28	0	
903	KFM04	One	Control	14	1	na
904	KFM04	One	Control	31	0	
905	KFM04	One	Control	31	0	
906	KFM04	One	Control	31	0	
907	KFM04	One	Control	31	0	
908	KFM04	One	Control	31	0	
909	KFM04	One	Control	31	0	
910	KFM04	One	Control	31	0	
911	KFM04	One	Control	31	0	
912	KFM04	One	Control	31	0	
913	KFM04	One	Control	31	0	
914	KFM04	One	Control	31	0	
915	KFM04	One	Control	31	0	
916	KFM04	One	Control	31	0	
917	KFM04	One	Control	31	0	
918	KFM04	One	Control	31	0	
919	KFM04	One	Control	31	0	
920	KFM04	One	Control	31	0	
921	KFM04	One	Control	31	0	

922	KFM04	One	Control	31	0	
923	KFM13	One	Control	13	1	na
924	KFM13	One	Control	17	1	
925	KFM13	One	Control	18	1	
926	KFM13	One	Control	22	1	
927	KFM13	One	Control	22	1	
928	KFM13	One	Control	25	1	
929	KFM13	One	Control	30	0	
930	KFM13	One	Control	30	0	
931	KFM13	One	Control	30	0	
932	KFM13	One	Control	30	0	
933	KFM13	One	Control	30	0	
934	KFM13	One	Control	30	0	
935	KFM13	One	Control	30	0	
936	KFM13	One	Control	30	0	
937	KFM13	One	Control	30	0	
938	KFM13	One	Control	30	0	
939	KFM13	One	Control	30	0	
940	KFM13	One	Control	30	0	
941	KFM13	One	Control	30	0	
942	KFM22	One	Control	2	1	na
943	KFM22	One	Control	17	1	
944	KFM22	One	Control	30	0	
945	KFM22	One	Control	30	0	
946	KFM22	One	Control	30	0	
947	KFM22	One	Control	30	0	
948	KFM22	One	Control	30	0	
949	KFM22	One	Control	30	0	
950	KFM22	One	Control	30	0	
951	KFM22	One	Control	30	0	
952	KFM22	One	Control	30	0	
953	KFM22	One	Control	30	0	
954	KFM22	One	Control	30	0	
955	KFM22	One	Control	30	0	
956	KFM22	One	Control	30	0	
957	KFM22	One	Control	30	0	
958	KFM22	One	Control	30	0	
959	KFM22	One	Control	30	0	
960	KFM22	One	Control	30	0	
961	KFM22	One	Control	30	0	
962	KFM26	One	Control	30	0	na
963	KFM26	One	Control	30	0	

964	KFM26	One	Control	30	0	
965	KFM26	One	Control	30	0	
966	KFM26	One	Control	30	0	
967	KFM26	One	Control	30	0	
968	KFM26	One	Control	30	0	
969	KFM26	One	Control	30	0	
970	KFM26	One	Control	30	0	
971	KFM26	One	Control	30	0	
972	KFM26	One	Control	30	0	
973	KFM26	One	Control	30	0	
974	KFM26	One	Control	30	0	
975	KFM26	One	Control	30	0	
976	KFM26	One	Control	30	0	
977	KFM26	One	Control	30	0	
978	KFM26	One	Control	30	0	
979	KFM26	One	Control	30	0	
980	KFM26	One	Control	30	0	
981	KFM26	One	Control	30	0	
982	KFM1	Open	Control	8	1	na
983	KFM1	Open	Control	23	1	
984	KFM1	Open	Control	25	1	
985	KFM1	Open	Control	28	0	
986	KFM1	Open	Control	28	0	
987	KFM1	Open	Control	28	0	
988	KFM1	Open	Control	28	0	
989	KFM1	Open	Control	28	0	
990	KFM1	Open	Control	28	0	
991	KFM1	Open	Control	28	0	
992	KFM1	Open	Control	28	0	
993	KFM1	Open	Control	28	0	
994	KFM1	Open	Control	28	0	
995	KFM1	Open	Control	28	0	
996	KFM1	Open	Control	28	0	
997	KFM1	Open	Control	28	0	
998	KFM1	Open	Control	28	0	
999	KFM1	Open	Control	28	0	
1000	KFM1	Open	Control	28	0	
1001	KFM1	Open	Control	28	0	
1002	KFM1	Open	Control	28	0	
