# Supplementary material for: Disease in the Society: Infectious Cadavers Result in Collapse of Ant Sub-Colonies
Source: PLoS One. 2016 Aug 16;11(8):e0160820. doi: 10.1371/journal.pone.0160820 (PMC4986943; doi:10.1371/journal.pone.0160820)
Supplement: S3 Table — (PDF) [file pone.0160820.s008.pdf]

Table S3: Data used to estimate the minimal number of ants directly infected by the introduced infectious cadaver

ID	Day of 1st death	Day of growth	% dead on day of growth	% dead day growth + day of 1st death
Flem11_TCC_B	4	6	82	100
Flem3_TCC_B	1	7	45	55
Flem5_TCC_B	4	9	45	100
Flem6_TCC_B	2	8	78	83
Flem7_TCC_B	1	7	65	95
KFM1_TCC_B	2	8	80	85
KFM16_TCC_B	3	6	62	90
KFM26_TCC_B	9	12	50	100
KFM4_TCC_B	4	10	40	70
Flem11_OCC_B	3	7	70	90
Flem3_OCC_B	4	7	76	100
Flem5_OCC_B	3	7	70	85
Flem6_OCC_B	3	7	95	100
Flem6_OCC_B	9	17	50	100
Flem8_OCC_B	4	7	75	100
KFM1_OCC_B	3	9	60	75
KFM11_OCC_B	4	7	60	75
KFM13_OCC_B	3	10	60	70
KFM16_OCC_B	4	8	71	100
KFM21_OCC_B	2	6	19	34
KFM22_OCC_B	4	7	70	85
KFM26_OCC_B	5	9	63	84
KFM4_OCC_B	1	12	30	30
